# Pharmacokinetic and pharmacodynamic characterization of gepotidacin against *Escherichia coli* and *Klebsiella pneumoniae* in a neutropenic mouse thigh infection model

**DOI:** 10.1128/aac.01176-25

**Published:** 2025-12-05

**Authors:** Christine M. Singley, Abhinav Kurumaddali, Jennifer L. Hoover

**Affiliations:** 1 GSK 525885 , Collegeville, Pennsylvania, USA; University of Houston, Houston, Texas, USA

**Keywords:** PK/PD, *in vivo*, gepotidacin, UTI

## Abstract

Gepotidacin is a novel first-in-class triazaacenaphthylene antibiotic developed for the treatment of uncomplicated gonorrhea and uncomplicated urinary tract infections (uUTIs). To support uUTI, *in vivo* pharmacokinetics (PK)/pharmacodynamics (PD) studies have been conducted in the murine neutropenic thigh infection model evaluating gepotidacin against 17 isolates of *Escherichia coli* and 7 isolates of *Klebsiella pneumoniae* having MICs of 0.25 to 16 µg/mL. Exposure data were fit using a population PK model, and efficacy data were fit with an inhibitory effect sigmoid Imax model using free-drug area under the concentration-time curve (fAUC)/MIC as the primary index. The ratios associated with response were determined for each isolate, and the median fAUC/MIC (excluding strains with <1-log of growth from baseline) was 13. To retain the data for all isolates, an exploratory analysis was conducted using different criteria to determine the target for strains with <1-log of growth (6 of the 24 strains tested). While the median fAUC/MIC was similar regardless of the criteria used, this analysis highlights the importance of critically reviewing PK/PD data for trends related to isolate characteristics and/or individual study outputs. As previously reported, the systemic targets determined from PK/PD studies were applied to urine concentrations for probability of target attainment analyses, which led to successful clinical trials and regulatory approval for gepotidacin in the treatment of uUTI. However, further work is needed to confirm the translational validity of these approaches on a broader scale and their application in establishing PK/PD targets for cystitis.

## INTRODUCTION

Uncomplicated urinary tract infections (uUTIs) and their recurrence affect more than 150 million women worldwide yearly and have a debilitating impact on the daily lives of those affected. The most common bacterial pathogen associated with uUTI is *Escherichia coli* (75% of cases) followed by *Klebsiella pneumoniae* (6% of cases) (1). A rise in antimicrobial resistance has made treatment more difficult, and few novel-acting agents have been developed for uUTI in the last several decades (2). Gepotidacin is an oral, first-in-class, triazaacenaphthylene antibacterial that selectively inhibits bacterial DNA gyrase and topoisomerase IV by a distinct binding site and a unique mechanism of action (3, 4). Gepotidacin has *in vitro* activity against target bacterial pathogens including *E. coli* and *K. pneumoniae,* the most prevalent causative pathogens of uUTI, including fluoroquinolone-resistant isolates (5, 6). It recently received FDA approval for the treatment of uUTI (7, 8), and a Phase 3 trial has also been completed assessing gepotidacin for efficacy and safety in the treatment of gonorrhea (9).

In the development of antibacterial treatments, characterization of pharmacokinetics (PK) and pharmacodynamics (PD) is required to inform appropriate dosing and to determine susceptibility breakpoints. Both a PK/PD index (such as area under the curve, maximum drug concentration, or the time that drug concentrations remain above the MIC) and a numerical “target” value for the index are needed. Efforts have been made to identify and promote best practice methods for the determination of PK/PD targets, including a workshop held in 2017 by the National Institute of Allergy and Infectious Diseases (10) and guidelines published by the Clinical & Laboratory Standards Institute (11). Guided by these proposed standards, PK/PD studies were conducted for gepotidacin against *E. coli* and *K. pneumoniae* (representing Enterobacterales as a group) in the well-established murine neutropenic thigh infection model. Here we report the results from these studies and the recommended *in vivo* PK/PD target for gepotidacin in the treatment of uUTI.

## RESULTS

### PK

**Fig 1:**
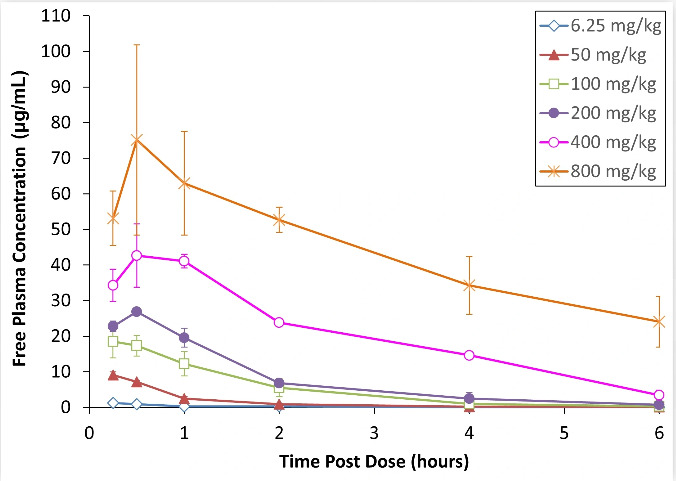
Observed free-drug plasma concentration-time profiles following subcutaneous dosing of gepotidacin to infected neutropenic mice. Data were pooled from all studies and all doses tested, and mean and standard deviations are indicated for each timepoint. *N* = 3 mice per study, dose, and timepoint with terminal sampling via cardiac puncture.

Following subcutaneous administration to infected neutropenic mice, gepotidacin exhibited a dose-dependent increase in exposure (Fig. 1), although a comparison of dose with free-drug peak plasma concentration (fCmax) and free-drug area under the concentration-time curve (fAUC)_0-6_ values indicated that exposure was slightly disproportional with dose (Table 1). This phenomenon was accounted for when building the mathematical models by incorporating dose as a covariate on the absorption rate constant. The final population PK model fit the data well, as evidenced by low %RSE on parameter estimates, even distribution of data points around the line of unity for observed vs estimated values, appropriate scattered distribution for conditionally weighted residuals, and normalized prediction distribution error vs time and vs predictions. Model details, diagnostics, and *post hoc* noncompartmental analysis (NCA) PK parameters are included in the Supplemental Materials (Fig. S1 through S4 and Tables S1 through S3).

**TABLE 1:** Observed free-drug Cmax and AUC_0-6_, pooled from all studies and doses tested following subcutaneous administration of gepotidacin to infected neutropenic mice

Dose (mg/kg)	fCmax^*a*^ (μg/mL)	fAUC_0-6_^*b*^ (μg h/mL)
6.25	1.21	1.27
50	9.04	8.58
100	18.5	30.7
200	26.9	46.2
400	42.7	124
800	75.1	260

^*a*^ Reflects the highest observed free-drug concentration from the composite PK profile (average of at least *N *= 3 mice per timepoint).

^*b*^ Free-drug area under the curve from time zero (where concentration was assumed to be zero) to the last timepoint measured (6 h post-dose) for the composite PK profile (average of at least *N *= 3 mice per timepoint).

### PD

The PD data were well fit by a 3-parameter inhibitory Imax model correlating exposure with change in bacterial burden (Fig. S5 through S9 and Table S4). Free-drug (f) AUC/MIC described the overall data set better (R2 = 0.61 and 0.80 for *E. coli* and *K. pneumoniae*, respectively) than the non-normalized parameter, fAUC (R2 = 0.35 and 0.70 for *E. coli* and *K. pneumoniae*, respectively) when all of the isolates were co-modeled together (Fig. S6 and S7 and Table S4). fAUC/MIC has also been shown previously to best characterize the exposure-response relationship for gepotidacin (12, 13) and, thus, only fAUC/MIC was considered as the PK/PD index for further analyses.

The fAUC/MIC ratios associated with stasis, a 1-log_10_ reduction, and 2-log_10_ reduction in colony-forming unit (CFU) for each isolate compared with its respective mean baseline control are summarized in Table 2, with strains that exhibited <1-log growth in vehicle-treated controls (VTCs) (compared with baseline) excluded from the results. As shown in Supplemental Materials, additional analyses were conducted with the inclusion of all isolates (Table S5 through S7); however, the results did not differ markedly from those shown in Table 2.

**TABLE 2:** fAUC/MIC targets against *E. coli* (EC) and *K. pneumoniae* (KP) from a thigh infection model in neutropenic mice

Strain^*a*^	MIC (µg/mL)	Change in VTC^*b*^	fAUC/MIC ratios		
			Stasis	1-log_10_ reduction	2-log_10_ reduction
EC Y6702902277A	0.25	+1.26	0.6	1.4	2.9
EC Y6702665868B	0.25	+2.04	7.7	18.3	37.9
EC Y6700050509B	0.5	+1.38	1.5	3.2	5.9
EC ATCC25922	1	+2.27	13.7	23.1	37.7
EC NCTC13441	2	+2.89	17.1	28.3	47.2
EC 997577	2	+2.48	10.5	18.4	32.5
EC ALL	4	+1.52	2.9	6.7	14.8
EC IR5	4	+2.05	2.5	4.9	10.2
EC 1139570	8	+1.28	2	4.8	9.8
EC 771034	8	+3.30	22.6	37.2	63
EC 764023	16	+2.96	1.2	2.2	4.4
EC 1032890	16	+2.94	21.4	29.9	39.5
KP 1478575	4	+2.06	5.3	10.4	20.6
KP 1478677	4	+1.73	3.1	5.7	10.1
KP 1203214	8	+2.37	5.6	10.4	19.4
KP 1449616	8	+1.21	2	5.6	11.6
KP 1511191	16	+1.26	1.1	2.8	6.6
KP 1511289	16	+3.22	5.9	9.5	15.6
Summary statistics for *E. coli* isolates					
Mean ± SD			8.6 ± 8.2	15 ± 13	25 ± 20
Median (range)			5.3 (0.6–23)	13 (1.4–37)	24 (2.9–63)
Summary statistics for *K. pneumoniae* isolates					
Mean ± SD			3.8 ± 2.0	7.4 ± 3.2	14 ± 5.5
Median (range)			4.2 (1.1–5.9)	7.6 (2.8–10)	14 (6.6–21)
Summary statistics for all isolates (representing Enterobacterales)					
Mean ± SD			7.0 ± 7.1	12 ± 11	22 ± 17
Median (range)			4.2 (0.6–23)	8.1 (1.4–37)	15 (2.9–63)

^*a*^ Strains with less than 1-log_10_ growth from baseline to end-of-study were excluded (see Tables S5 and S6 for all strains).

^*b*^ Average change in CFU at end of study in VTC mice compared with the average baseline CFU in untreated mice at 1 h post-infection.

**Fig 2:**
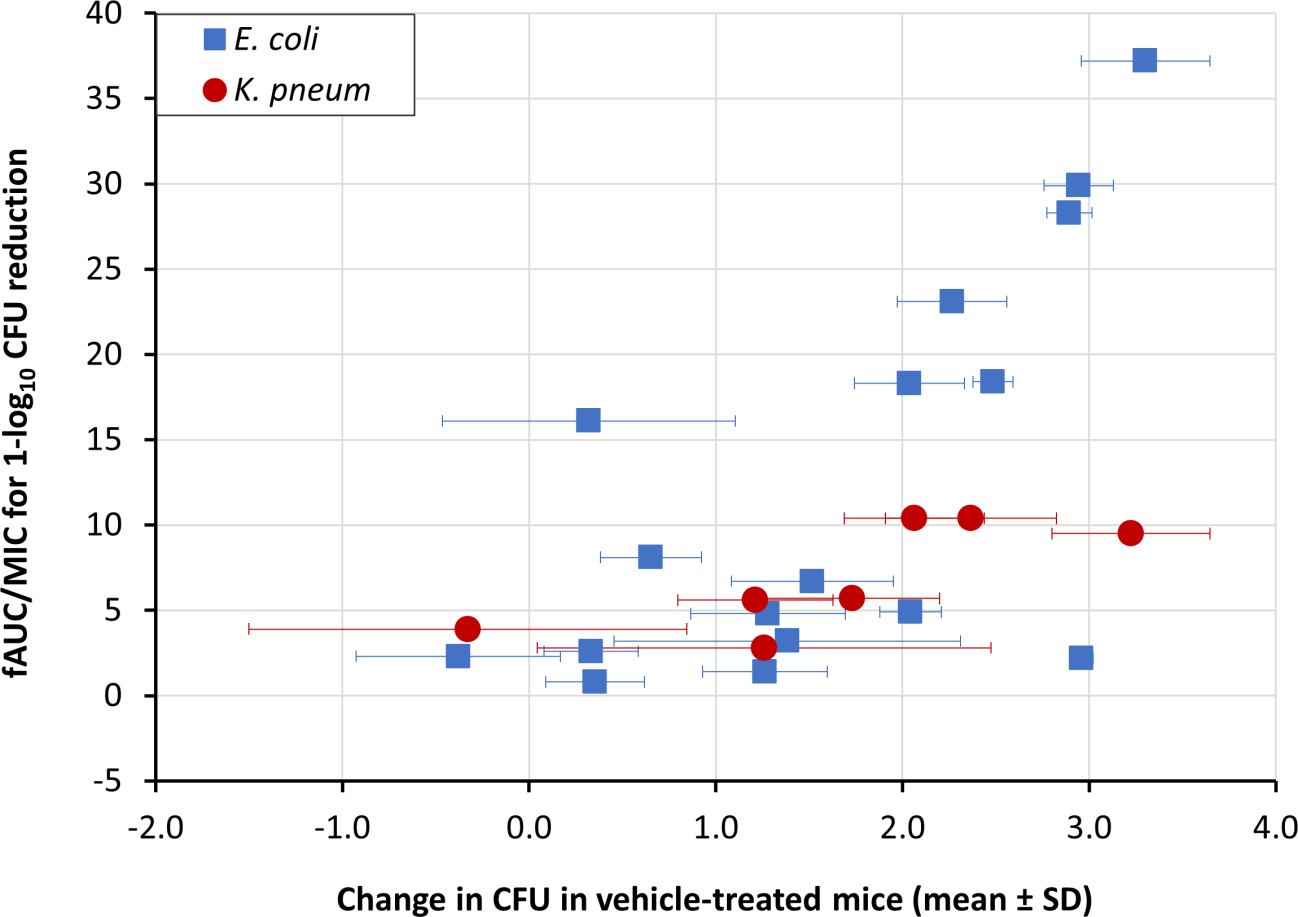
Relationship between bacterial growth and gepotidacin fAUC/MIC ratio. Data points represent the growth of individual isolates (squares for *E. coli* and circles for *K. pneumoniae*) shown as mean ± standard deviation change in CFU in vehicle-treated mice (*N* = 5) vs mean baseline CFU. Points with no visible error bars indicate that CFUs from all mice in the group were too numerous to count and were assigned the upper limit of quantification. A nonsignificant trend was observed whereby some isolates with more growth required a higher fAUC/MIC ratio to achieve a 1-log_10_ reduction in CFU.

There appeared to be some correlation between the fAUC/MIC ratio required for a 1-log_10_ reduction in CFU and performance of the bacterial isolates in the model. A nonsignificant trend was observed whereby some isolates with more growth in vehicle-treated mice required higher fAUC/MIC ratios for efficacy (Fig. 2). To prevent bias toward a lower overall target value by including strains that had less growth, the primary analysis shown in Table 2 excluded those that did not grow at least 1-log_10_ vs baseline. However, this resulted in a loss of information, particularly for isolates with higher MIC values, which were considered important for understanding the efficacy of gepotidacin across the range of the MIC distribution. Thus, additional exploratory analyses were conducted to assess the impact of including all the data by calculating summary statistics as follows: (i) inclusion of all strains using the same endpoint regardless of growth and (ii) incorporating all isolates but using the 2-log_10_ reduction target value (instead of 1-log_10_ reduction) for those isolates demonstrating <1-log_10_ growth in the model. These exploratory results are shown in Tables S5 through S7.

For infections considered to have a high organism burden and low spontaneous resolution rate, an endpoint of at least 1-log_10_ reduction in CFU in the neutropenic thigh infection model is recommended (11). There is no consensus on whether uUTI aligns with this description, and it may be appropriate to consider a stasis target for this indication. However, the PK/PD target associated with a 1-log_10_ reduction in CFU was prioritized for gepotidacin as per regulatory advice. While the overall median values for both stasis and a 1-log reduction were lower for *K. pneumoniae* (4.2 and 7.6, respectively) than *E. coli* (5.3 and 13, respectively), the higher of the targets was selected to ensure coverage of both species. Thus, using these criteria, a fAUC/MIC ratio of 13 is the *in vivo* PK/PD target for gepotidacin against Enterobacterales.

## DISCUSSION

These studies were undertaken to characterize the PK/PD of gepotidacin against *E. coli* and *K. pneumoniae* using a thigh infection model in neutropenic mice and a large panel of challenging isolates with gepotidacin MICs up to 16 μg/mL (Table 3). Strains were selected based on the MIC distribution for *E. coli* and *K. pneumoniae*, including those designated FQ-resistant, MDR, and/or producing ESBL and MBL enzymes, and encompassed the MIC90 (4 and 16 μg/mL, respectively) as determined from isolates collected in two global, large-scale uUTI trials (6). From these studies, a fAUC/MIC of 13 was the target exposure required to achieve a 1-log_10_ reduction in the neutropenic mouse thigh infection model against the two most prevalent bacterial species implicated in uUTI.

**TABLE 3:** Characteristics of challenge isolates used in these studies^*a*^

Species	Strain	Gepotidacin MIC (µg/mL)	Antibiotic resistance
*E. coli*	Y6702902277A	0.25	AMP
*E. coli*	Y6702665868B	0.25	AMP, FQ, SXT
*E. coli*	Y6700050509B	0.5	FQ, SXT
*E. coli*	Y6700034261B	0.5	FQ, SXT
*E. coli*	ATCC25922	1	No resistance reported
*E. coli*	NCTC13441	2	Not specified; produces ESBL (CTX-M-15) and harbors sequenced plasmid pEK499
*E. coli*	997577	2	SAM, ATM, AMG, Cephems, Carbapenems, FQ; produces ESBLs (TEM-OSBL(b), CTX-M-15) and NDM-5
*E. coli*	ALL	4	Cephems, FQ, Carbapenems; produces NDM-1
*E. coli*	IR5	4	Cephems, FQ, Carbapenems; produces ESBLs (CTM-X-15, TEM-1) and NDM-1
*E. coli*	1139570	8	SAM, AMG, Cephems, Carbapenems; produces ESBL (TEM-OSBL(b)) and NDM-1
*E. coli*	774319	8	No resistance reported
*E. coli*	817317	8	FQ
*E. coli*	771034	8	No resistance reported
*E. coli*	764023	16	No resistance reported
*E. coli*	1032890	16	SAM, ATM, Cephems, FQ, Carbapenems; produces ESBL (CTX-M-3) and NDM-1
*E. coli*	823196	16	FQ; MDR
*E. coli*	718513	16	No resistance reported
*K. pneumoniae*	1286210	2	FQ, PIP; produces ESBLs (SHV-OSBL, TEM-OSBL), AmpC, and DHA-1
*K. pneumoniae*	1478575	4	No resistance reported
*K. pneumoniae*	1478677	4	No resistance reported
*K. pneumoniae*	1203214	8	CEF, TAZ, MER, IMI; produces ESBL (SHV-OSBL), MBL, and VIM-1
*K. pneumoniae*	1449616	8	No resistance reported
*K. pneumoniae*	1511191	16	FQ, SXT
*K. pneumoniae*	1511289	16	Cephems, FQ, SXT

^*a*^ AMP, ampicillin; SAM, ampicillin/sulbactam; FQ, fluoroquinolone; SXT, trimethoprim/sulfamethoxazole; ATM, aztreonam; AMG, aminoglycoside; MDR, multi-drug resistant; PIP, piperacillin; CEF, cefepime; TAZ, ceftazidime; MER, meropenem; IMI, imipenem; ESBL, extended-spectrum beta-lactamase; NDM, New Delhi metallo-beta-lactamase; AmpC, AmpC beta-lactamase; DHA-1, a class C beta-lactamase; MBL, metallo-beta-lactamase; VIM-1, a metallo-beta-lactamase.

A nonsignificant trend was observed that more drug was required for isolates that grew better in the model. This was unexpected; however, the link between bacterial growth and susceptibility to antibiotics has been reported and investigated by others (12, 14–16). This trend was not seen with all isolates, and other than strain growth, there was no other parameter that correlated with the outcome including starting inoculum, baseline bacterial burden, severity of disease, and fluoroquinolone resistance (data not shown). To ensure the outcome was not biased toward a lower target, the results for isolates that showed less than 1-log_10_ growth in the model were excluded from the primary output. However, as an exploratory approach to retain the data for all isolates (which were purposefully selected to assess gepotidacin’s efficacy against strains with specific characteristics and MIC values), the summary statistics were adjusted by using the fAUC/MIC associated with 2-log_10_ of killing for those isolates with less than 1-log_10_ of growth in the model (*N* = 6 among the 24 total isolates tested) (Tables S5 through S7). Additional and/or more complex analyses could be explored to account for the potential effect of growth on PD response; for example, the PK/PD target for each strain could be normalized by its corresponding growth profile. However, in this case, as the median PK/PD target applying this strategy was similar to the target determined by excluding strains that did not grow 1-log, a further analysis was not undertaken since it was expected to have little impact on the outcome.

Although some investigators have explored how the kinetics of bacterial growth impact antibacterial efficacy (14–16), it is not known how strain growth impacts PK/PD targets determined in the neutropenic thigh infection model. The standard interpretive approach applies the same criteria to all strains regardless of the speed or magnitude of their growth (i.e., whether a strain grows 1-log or 3-logs and/or causes rapid morbidity is not considered in the outputs). In this work, strains that showed spontaneous resolution of infection (defined as a statistically significant reduction in mean CFU vs baseline) were removed from consideration and are not included in the data presented here. However, strains that were not significantly lower than baseline but showed less than 1-log of growth at the end of the study were retained (as they were considered to provide valuable information), and exploratory analyses using a more stringent endpoint for these isolates were undertaken in an effort to account for the impact of strain growth. The translational validity of this approach is unproven, but it emphasizes the importance of considering strain behavior when interpreting PK/PD results. Further work is needed to elucidate how strain performance impacts the final PK/PD target and to guide investigators in appropriately accounting for strain differences in growth and lethality.

A common limitation of PK/PD studies, particularly in mice, is that PK sampling is generally a terminal procedure (due to the blood volume required per sample). Given the sparse sampling, we observed high epsilon-shrinkage (i.e., individual predictions shrink toward observed dependent variable, and the residual error, epsilon, shrinks toward 0). While individual PK profiles could not be determined per subject (e.g., mouse), for simulating mean PK profiles, this modeling approach was applied despite high epsilon-shrinkage (17) assuming that residual variability is negligible and/or is similar across the individual subjects (e.g., mouse).

It is generally recommended that nonclinical PK/PD studies be conducted using an animal model that most closely represents the human infection (18). However, for uUTI, there are important limitations among the available animal models, including the most commonly used murine cystitis model (19, 20). In addition to differences in host factors between mice and humans, we and others have found that the infection in mice tends to be transient and frequently self-resolves. While some bacterial isolates are virulent in the cystitis model, there are many isolates that do not grow and/or that show spontaneous resolution with no treatment. Few, if any, antibacterials have utilized the mouse cystitis model to establish PK/PD targets for clinical infection, leading to a scarcity of information regarding its clinical translatability. Owing to concerns that the cystitis model would potentially underestimate the amount of drug required for clinical efficacy and the limitation on the number of isolates that could be studied in the model, we chose to utilize the “gold standard” murine neutropenic thigh infection model (10, 11) to elucidate *in vivo* PK/PD targets for *E. coli* and *K. pneumoniae*.

As described in Scangarella-Oman et al. (21), the human dose prediction for gepotidacin in uUTI was based on the probability of achieving the designated PK/PD target in urine rather than blood. For the reasons described above, we did not conduct *in vivo* nonclinical uUTI models to corroborate this approach. However, once an antibacterial PK/PD target has been established for a given bug-drug combination, it may be reasonable to assume that achieving the same free-drug exposure at another site of infection should provide similar efficacy. Therefore, we applied the systemic fAUC/MIC target derived using the thigh infection model to urine exposures from patients to determine the probability of target attainment (PTA) in patients with uUTI (21). This translational extension assumes that the physiological differences between infection occurring in the thigh vs bladder do not impact antibacterial activity. However, as noted above, existing mouse bladder infection models were considered too limited and too permissive for determining reliable gepotidacin PK/PD targets, and thus, the clinically validated neutropenic thigh infection model was utilized rather than a mouse bladder infection model. Application of the *in vivo*-derived PK/PD targets (from the thigh infection model) and *in vitro*-derived targets (from the one-compartment chemostat model) to determine PTAs in uUTI (21) has been a successful approach for gepotidacin, evidenced by positive patient outcomes from two large-scale clinical trials (8), the subsequent approval of gepotidacin for the treatment of uUTI (7), and recognition of a gepotidacin Enterobacterales susceptibility breakpoint of ≤16 μg/mL in the US (22). It is not known if this translational approach can be applied more broadly to other compounds. Similar analyses conducted for additional antibacterials would be informative to fill the knowledge gap for the application of PK/PD principles in the treatment of uUTI.

## MATERIALS AND METHODS

### Bacterial isolates

All 24 isolates used in these studies were either clinical isolates obtained from the GSK culture collection, American Type Culture Collection (ATCC), or National Collection of Type Cultures (NCTC). A total of 24 isolates were used—17 isolates of *E. coli* and 7 isolates of *K. pneumoniae*—including MDR isolates, with gepotidacin MICs of 0.25–16 µg/mL (Table 3). MICs were determined in at least triplicate using broth microdilution as per CLSI methodology, and the modal values were used as the final MIC for each strain. Strains were selected to encompass the MIC distribution range for gepotidacin, and they included isolates which represent the MIC90 for *E. coli* and *K. pneumoniae* (4 and 16 μg/mL, respectively, as determined from isolates collected in two global, large-scale clinical trials [6]); furthermore, a sufficient number of isolates at the higher MIC values for both species were considered necessary to provide supporting information for establishment of a single susceptibility breakpoint for Enterobacterales.

Frozen stocks were cultured overnight with gentle agitation in brain heart infusion (BHI) broth at 37°C in ambient air. On the day of infection, a fresh log phase culture was prepared from the overnight broth by subculturing a 1 mL aliquot in 50 mL BHI for 3 h. The culture was centrifuged for 5 min at 4,200 rpm (4,122 × *g*), and the pellet was resuspended in sterile saline. This was repeated two more times, and the final suspension was diluted using a predetermined ratio of suspension to diluent for each isolate to achieve the proper inoculum for infection (ranging 7–8.5 CFU/mL). The inoculum required for each strain was evaluated in pilot studies and individualized to ensure a baseline of 6–7-log_10_ CFU/tissue was achieved at 1 h post-infection, which is within the recommended range as per published guidelines (10).

### Animals

Specific pathogen-free male CD-1 mice (Charles River, Raleigh, North Carolina) weighing 27–28 g were used in all experiments. All studies were conducted in accordance with the GSK Policy on the Care, Welfare and Treatment of Laboratory Animals and were reviewed by the Institutional Animal Care and Use Committee at GSK. In most studies, the mice were randomized by body weight prior to study start; note this did not occur in all studies because the practice was adopted at the testing facility after experiments had been initiated. Groups consisted of three animals for the PK studies and five for the efficacy studies based on statistical review and historical precedent. Groups were not blinded for infection or dosing; however, the primary readout (bacterial counts) was blinded to the study investigator. For all experiments, mice were rendered neutropenic (i.e., having no more than 100 polymorphonuclear leukocytes/mm^3^) by two 0.5 mL intraperitoneal injections of cyclophosphamide (Baxter, Deerfield, IL, USA), consisting of 150 mg/kg on day −4 and 100 mg/kg on day −1 pre-infection (23). Mice were housed 3–5 per box on alpha dry bedding with standard enrichment with standard 12 h light/dark cycles, room temperature of 68°F–79°F, and humidity of 30%–70%. They were allowed access to Lab DIET-Rodent diet 5001 food and bottled water *ad libitum*.

### Infection

Mice were briefly restrained, and one thigh was infected by injecting 0.1 mL of the prepared bacterial suspension into the muscle. Only one thigh was infected, yielding *N* = 1 result per mouse. Final inocula were between 6.4 and 8.1-log_10_ CFU/mouse, with the inoculum tailored for each isolate to achieve a 1 h baseline count of approximately 6.5-log_10_ CFU/mouse.

### For PK studies

Infected, neutropenic mice were administered a single dose of gepotidacin as a solution in sterile saline (6.25–800 mg/kg in a volume of 0.2 mL/mouse) at 1 h post-infection by subcutaneous injection. Terminal plasma samples were collected at 0.25, 0.5, 1, 2, 4, and 6 h post-dose. Approximately 500 µL of whole blood was collected via cardiac puncture and immediately placed into microfuge tubes coated with K2-EDTA. Blood was centrifuged for approximately 30 s at 12,000 rpm to separate plasma. Aliquots of 50 µL plasma were placed on dry ice immediately following centrifugation and maintained at −80°C until analysis using HPLC-tandem mass spectroscopy (MS/MS) with electrospray ionization working in multiple-reaction-monitoring mode with a Waters Acquity ultrahigh-performance liquid chromatograph connected to an API Sciex 4000 tandem quadrupole mass spectrometer. The lower limit of quantification for gepotidacin in mouse plasma was 0.05 µg/mL.

### For PD studies

Gepotidacin was prepared as described for the PK studies, and mice were administered doses ranging from 1 to 600 mg/kg by subcutaneous injection into the scruff of the neck in a volume of 0.2 mL/mouse starting at 1 h post-infection and continuing every 6 h (Q6) for 24 h (a total of four doses administered). One group of animals was euthanized by carbon dioxide overdose at 1 h post-infection to determine bacterial numbers present in the thigh at the time of treatment initiation; all remaining animals were euthanized at 25 h post-infection (approximately 6 h following the last administered dose). The entire thigh muscle was excised aseptically and homogenized in 1 mL sterile saline using a laboratory blender (Stomacher 80, Seward Ltd., Worthing, UK). Ten-fold serial dilutions of the tissue homogenates were prepared in saline, and the samples were inoculated in triplicate of 20 µL each onto trypticase soy agar plates supplemented with 5% sheep’s blood by a modified Miles Misra technique using the STAR liquid handling system (Hamilton). The plates were blinded, and colonies were counted by visual inspection following overnight incubation at 37°C. The lower and upper limits of quantification were 1.2 and 9.4-log_10_ CFU/thigh, respectively.

### Mathematical modeling

A population PK model was developed using NONMEM (v7.3), and the software R (v3.2.5) was used to create diagnostic plots (Fig. S2 through S4). The PK study design allowed for the collection of only one PK sample per mouse at different time intervals post-dose (0.25, 0.5, 1, 2, 4, and 6 h). Therefore, a simulation was performed with the final PopPK model using the mrgsolve package in R software (v3.2.5) to generate the full concentration versus time profiles (0–24 h PK profile including multiple doses administered every 6 h) at the different dose levels tested in the PD dose ranging efficacy studies. The total drug concentrations were converted to free-drug (f) values based on the protein binding value of 24% bound in mouse plasma (12). Gepotidacin has not exhibited concentration‐dependent protein binding in any species; therefore, a single point estimate is considered appropriate to correct for its plasma protein binding. The following parameters were computed via NCA using R software: fCmax, fAUCs from time 0–24 h for repeat dosing every 6 h (incorporating residual concentrations before administration of next dose), and the time (in hours) over which free-drug concentrations remained above a specific MIC value (fT > MIC).

The PD outcome measured from the dose-ranging efficacy studies was the number of bacteria isolated from the thigh expressed as log_10_ CFU. It was not feasible to measure PK and PD outcomes simultaneously in the same group of mice. Therefore, the relationship between the change in log_10_ CFU for each animal at the end of the study compared with the average baseline CFU from 1 h nontreated control group and the corresponding plasma PK derived from the final Pop PK model was modeled using a 3-parameter inhibitory Imax model (Fig. S5 through S9). The fAUC/MIC associated with stasis, 1-log_10_, or 2-log_10_ reductions in CFU were determined for each strain. The 3-parameter model and fAUC/MIC were selected for analysis of the data for individual bacterial isolates, and summary statistics were calculated per bacterial species and for all isolates combined into a single data set (representing Enterobacterales).

## SUPPLEMENTAL MATERIAL

Supplemental material aac.01176-25-s0001.docx Fig. S1 to S9; Tables S1 to S7.

ASM does not own the copyrights to Supplemental Material that may be linked to, or accessed through, an article. The authors have granted ASM a non-exclusive, world-wide license to publish the Supplemental Material files. Please contact the corresponding author directly for reuse.
