# Supplementary material for: Pharmacokinetic and pharmacodynamic characterization of gepotidacin against Escherichia coli and Klebsiella pneumoniae in a neutropenic mouse thigh infection model
Source: Antimicrob Agents Chemother. 2025 Dec 5;70(1):e01176-25. doi: 10.1128/aac.01176-25 (PMC12777569; doi:10.1128/aac.01176-25)
Supplement: Supplemental material — Fig. S1 to S9; Tables S1 to S7. [file aac.01176-25-s0001.docx]

**SUPPLEMENTARY MATERIAL**

Pharmacokinetic and pharmacodynamic characterization of gepotidacin against *Escherichia coli* and *Klebsiella pneumoniae* in a neutropenic mouse thigh infection model

**FIGURES:**

**Figure S1**: One compartment PK model schematic:


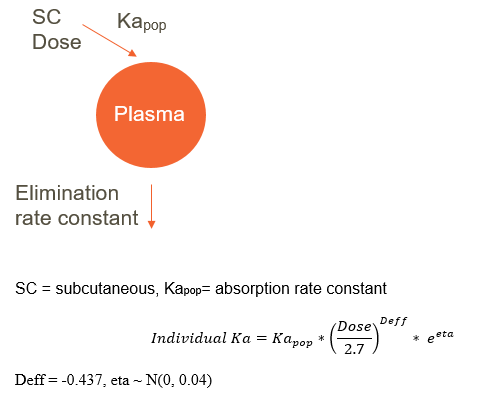


**Figure S2:** PK model diagnostics: Dependent variable (DV) versus prediction


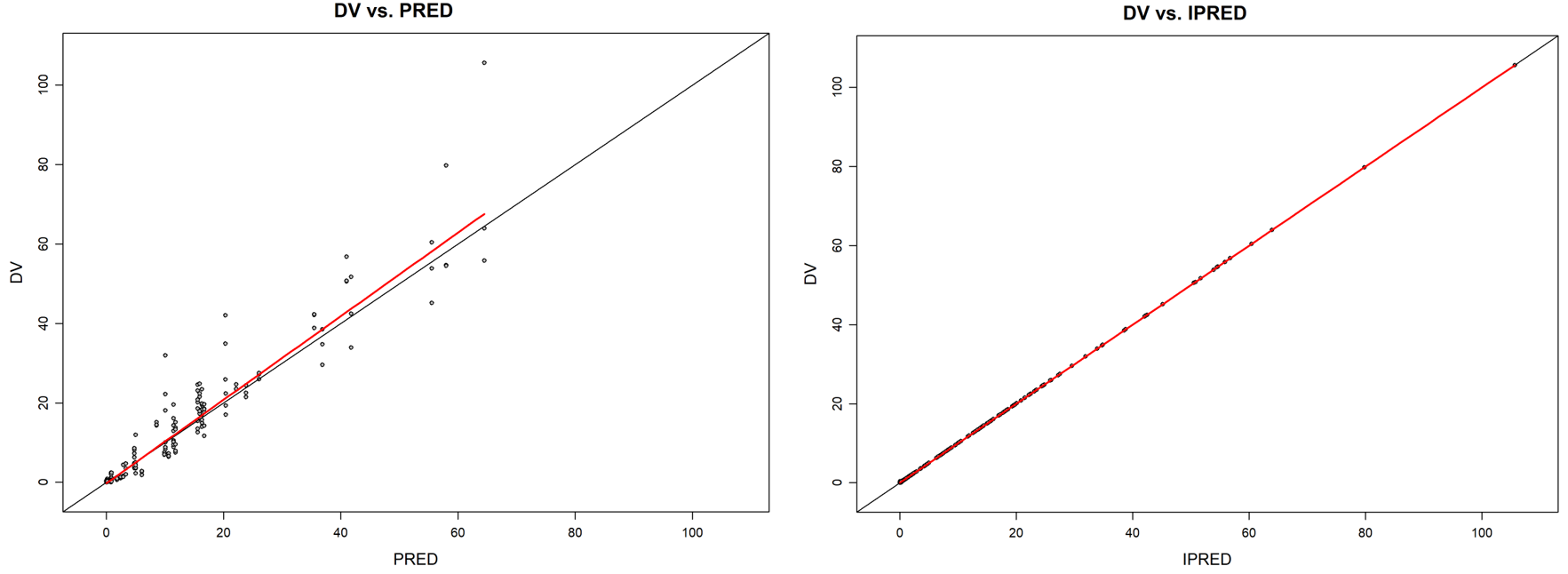


**Figure S3:** PK model diagnostics: Conditionally weighted residuals (CWRES) versus time and prediction


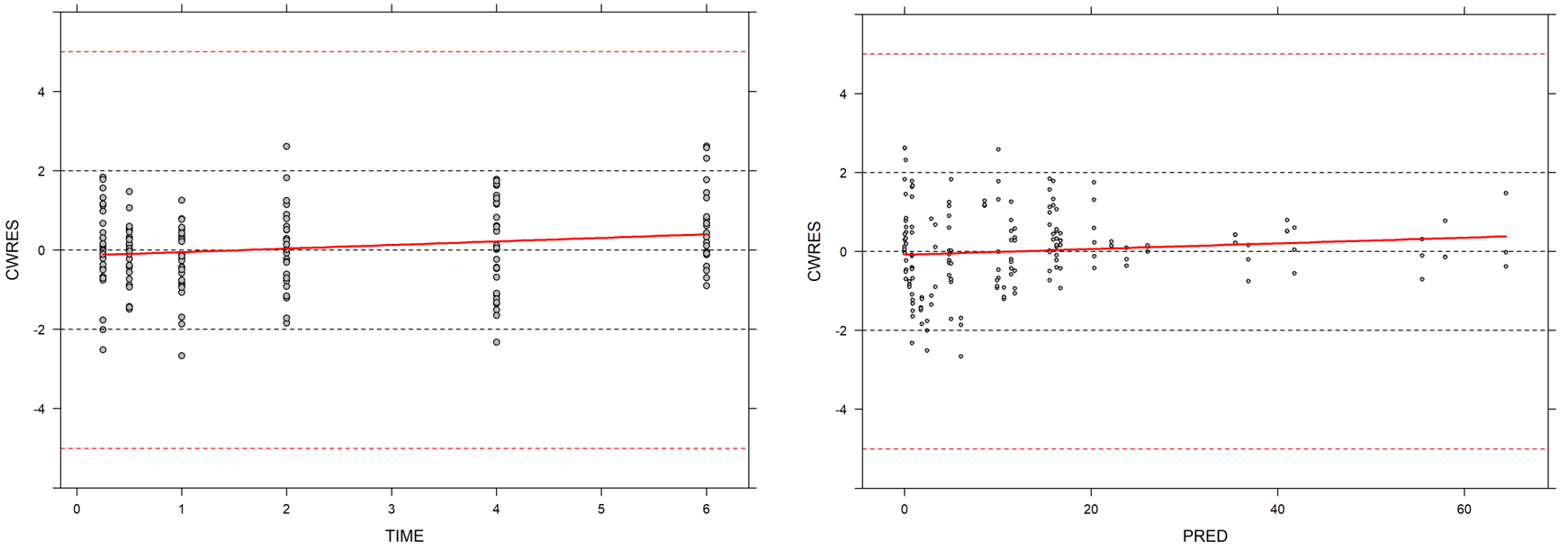


**Figure S4:**  PK model diagnostics: Normalized prediction distribution error (NPDE) versus time and prediction


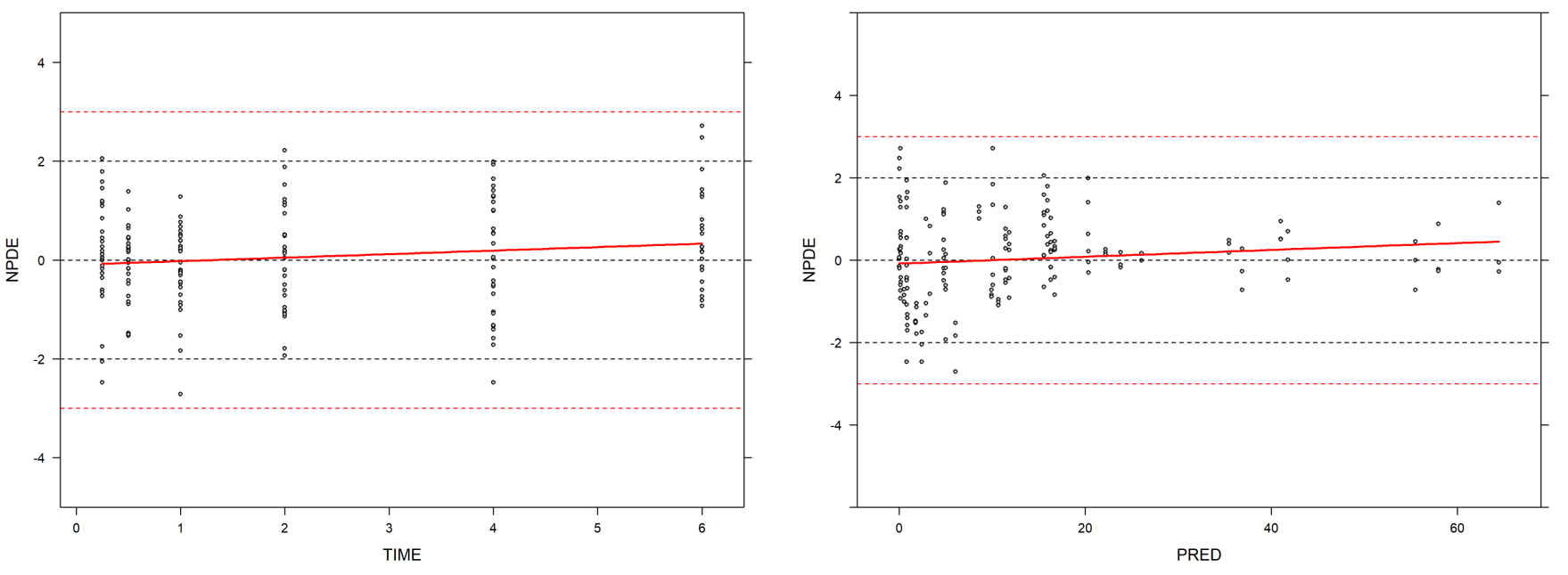


**Figure S5:** Inhibitory effect sigmoid Imax model used for analysis of PK/PD data

Where Effect (E) is the change in log_10_ CFU at the end of the study compared with baseline controls at the start of treatment (ΔCFU); E0 is the ΔCFU for vehicle-treated controls; Imax is the maximum change in CFU between vehicle-treated controls and the highest treatment dose tested; and IC50 is the PK/PD index value (e.g., fAUC/MIC) required to produce 50% of the Imax. In this analysis, the ΔCFU (i.e., Effect) vs. fAUC/MIC or fAUC (i.e., PK/PD Index) were modelled individually for each bacterial isolate and as a compiled dataset including all isolates of a given species (i.e., all *E. coli* isolates together and all *K. pneumoniae* isolates together) to determine the exposure required for a specified effect endpoint (i.e., no change, a 1-log_10_ reduction or a 2-log_10_ reduction in CFU).

**Figure S6:**  All *E. coli* Isolates (N=17) and Studies Co-Modelled


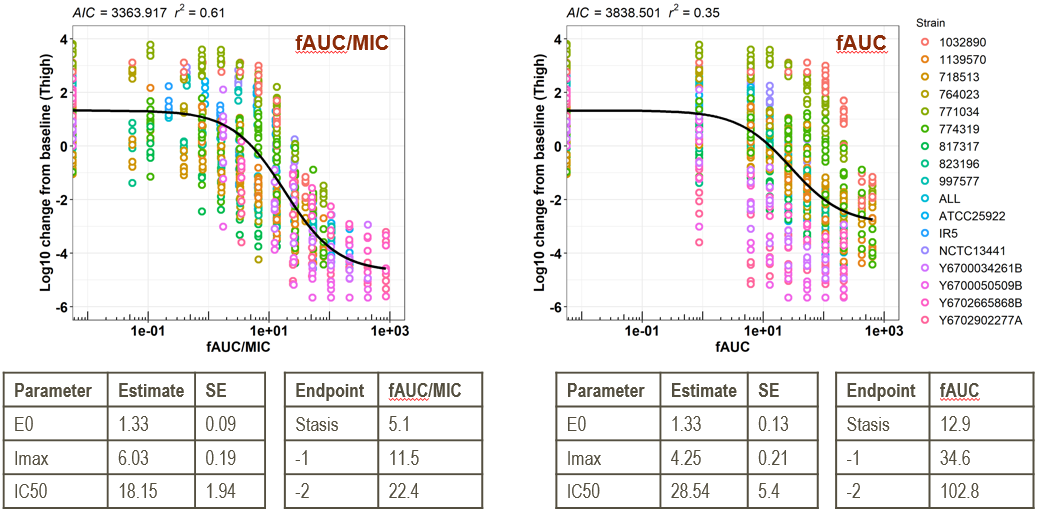


**Figure S7:** All *K. pneumoniae* Isolates (N=7) and Studies Co-Modelled


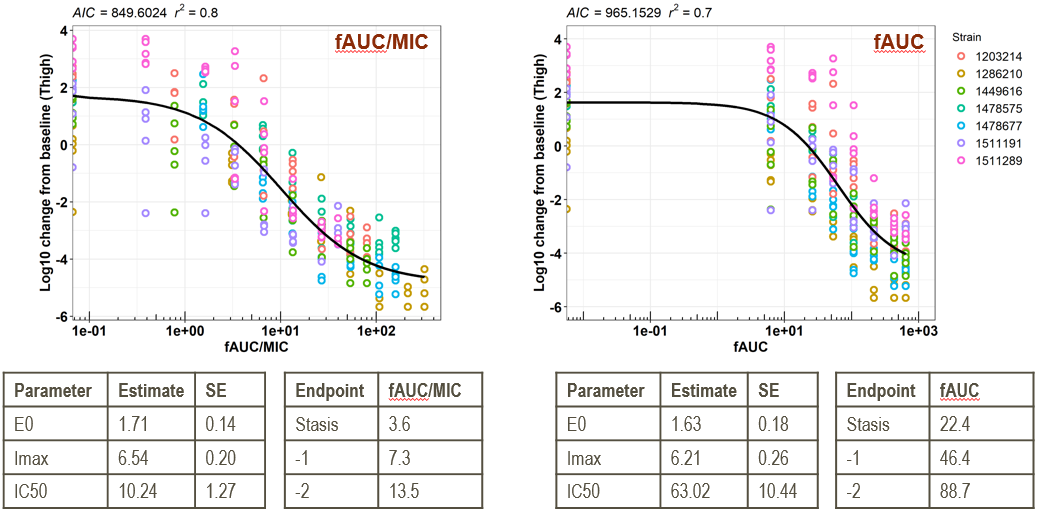


*Note that R2 values were 0.80 and 0.70 for fAUC/MIC and fAUC, respectively (see Table S4).*

**Figure S8:** Exposure-response relationships between fAUC/MIC ratio or fAUC (not normalized by MIC) for gepotidacin and change in bacterial burden from baseline for 17 *E. coli* isolates tested in a neutropenic thigh infection model in mice; colored symbols represent datapoints for individual mice; solid line represents the fitted model with tabulated model parameters, diagnostics and calculated PK/PD targets; some isolates were tested in multiple studies, and only the pooled data was analyzed

*E. coli* Y6702902277A


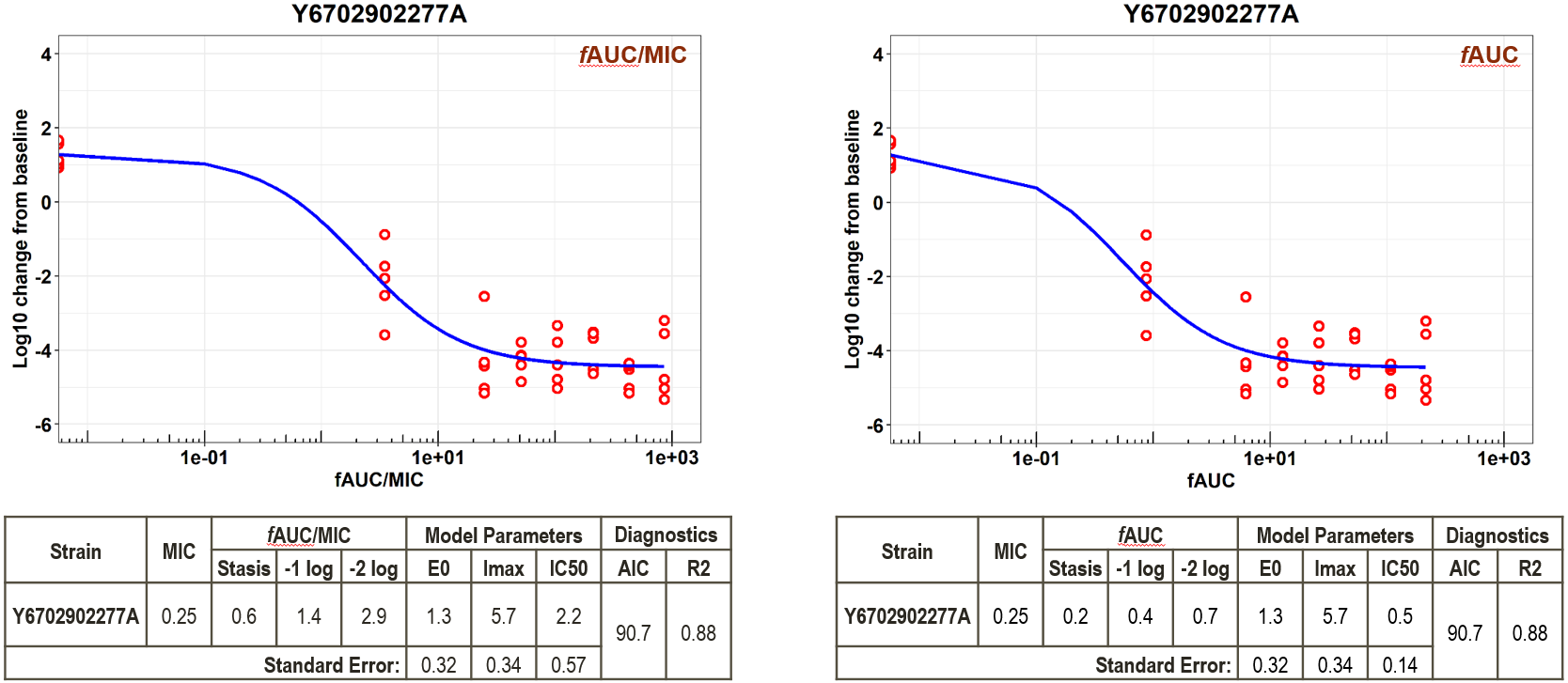


*E. coli* Y6702665868B


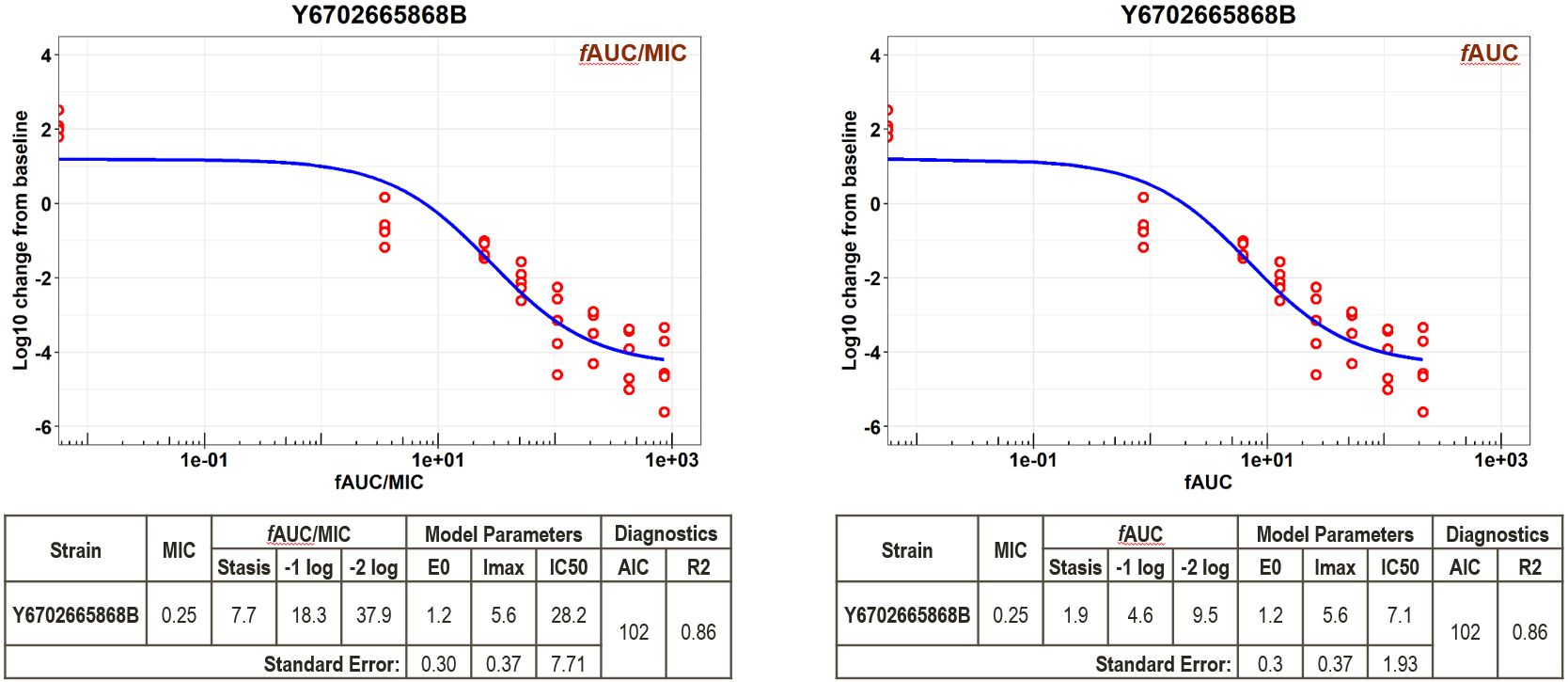


*E. coli* Y6700050509B


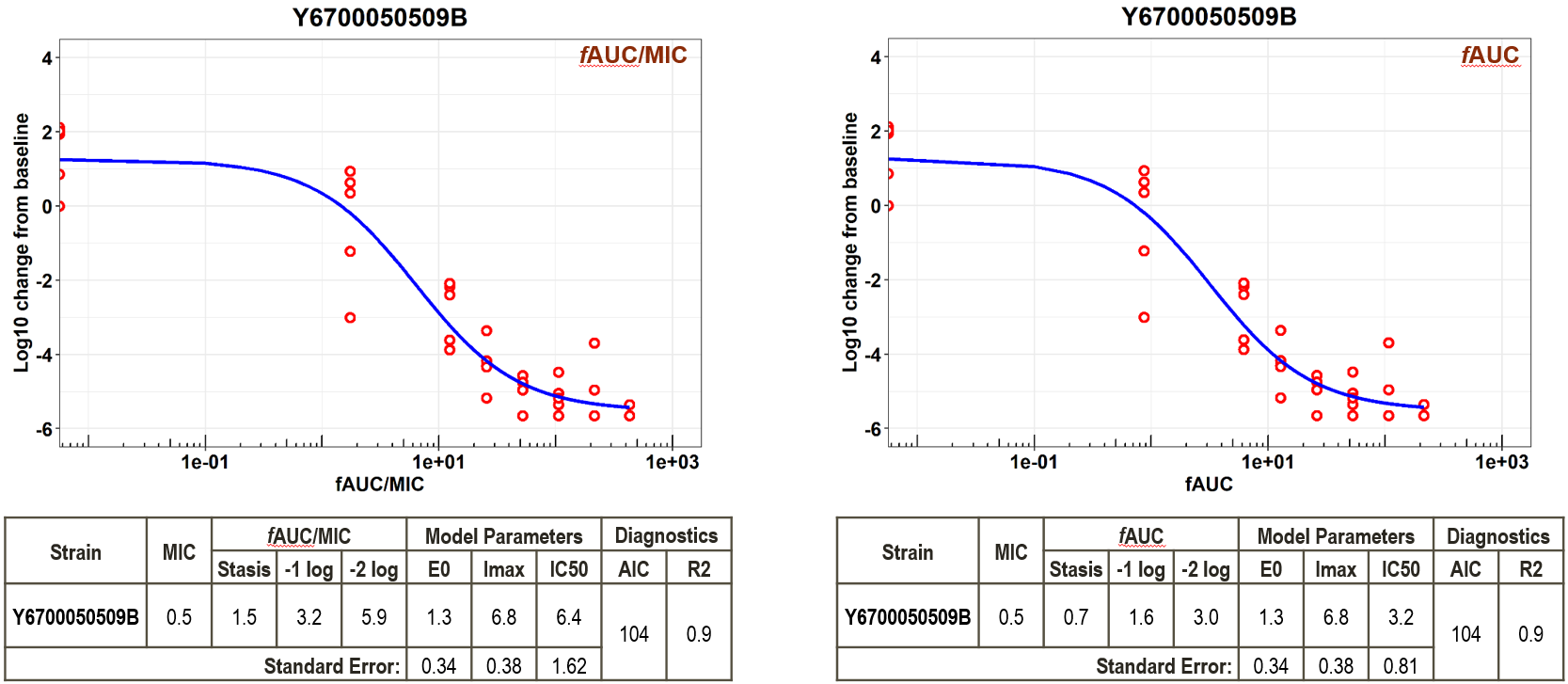


*E. coli* Y6700034261B


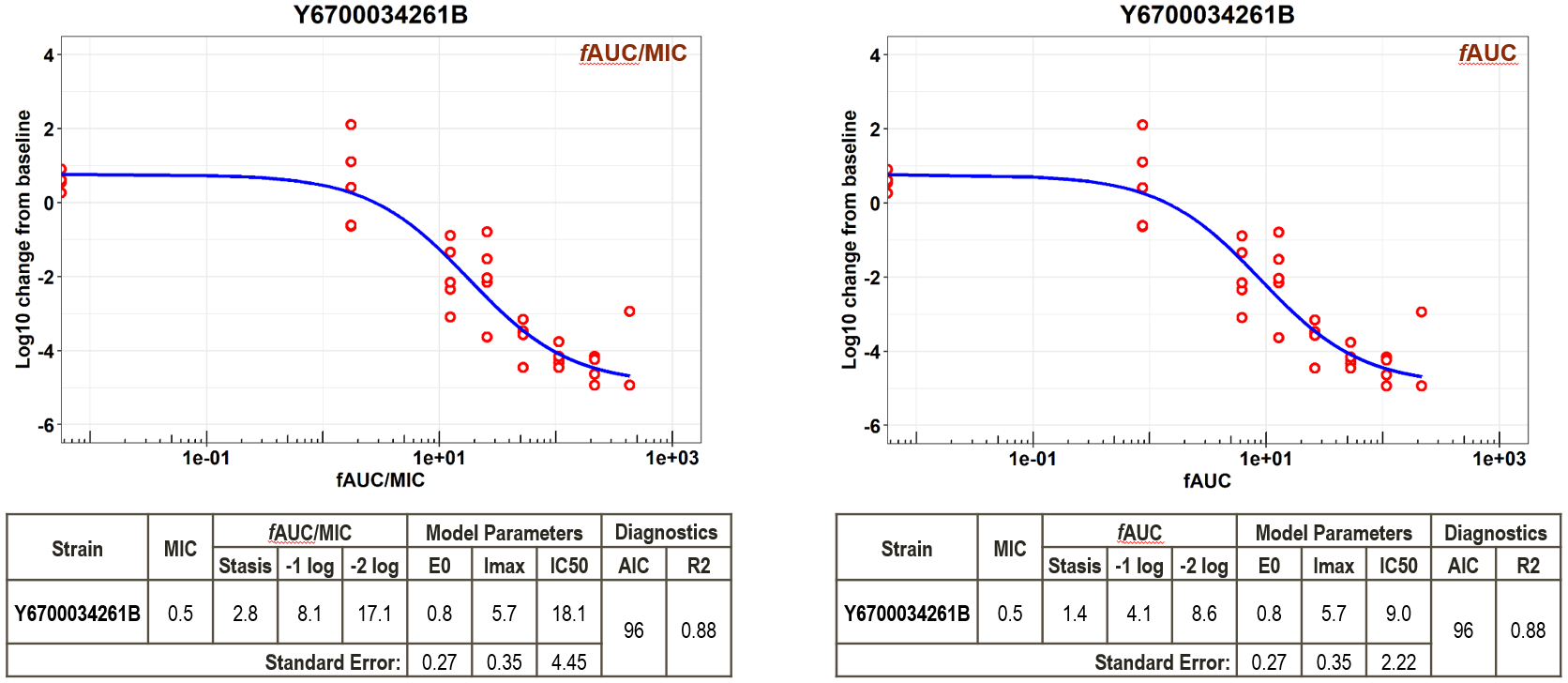


*E. coli* ATCC25922


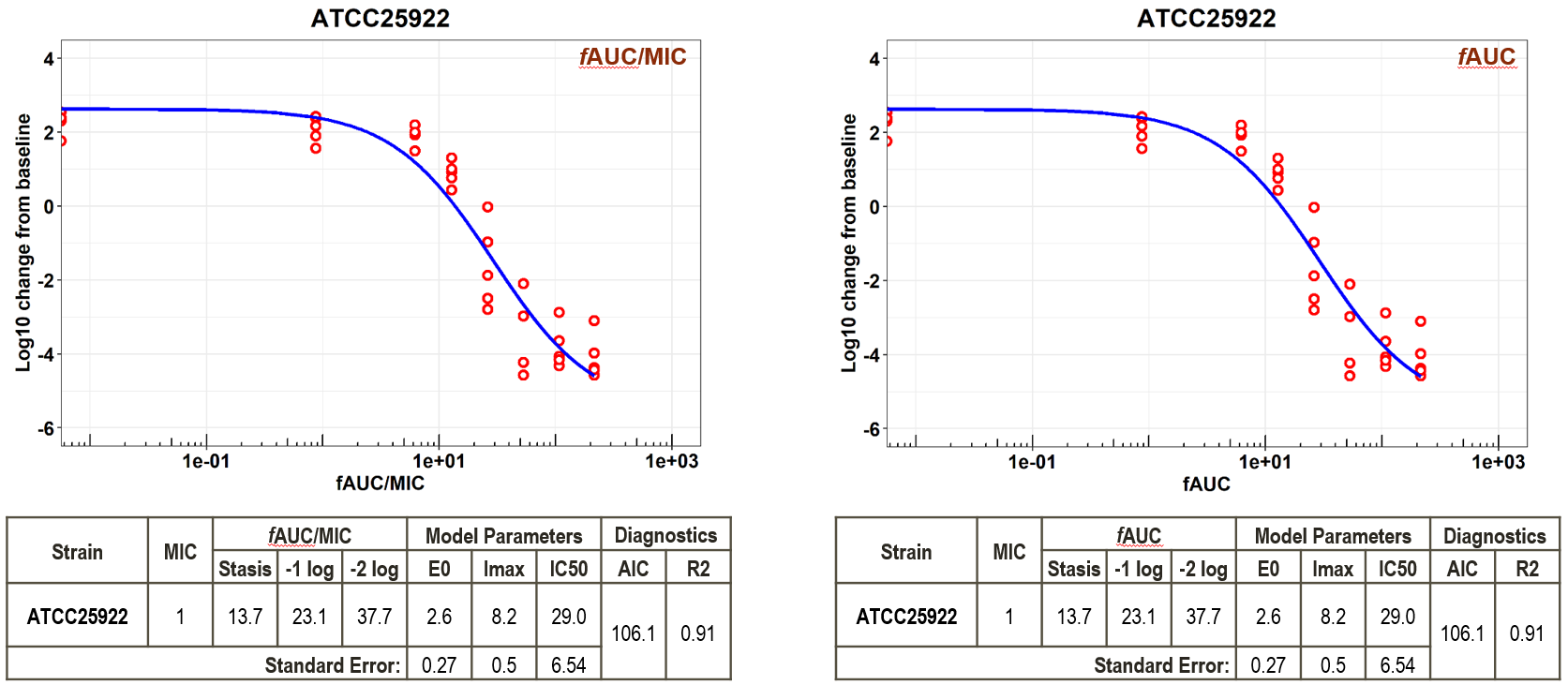


*E. coli* NCTC13441


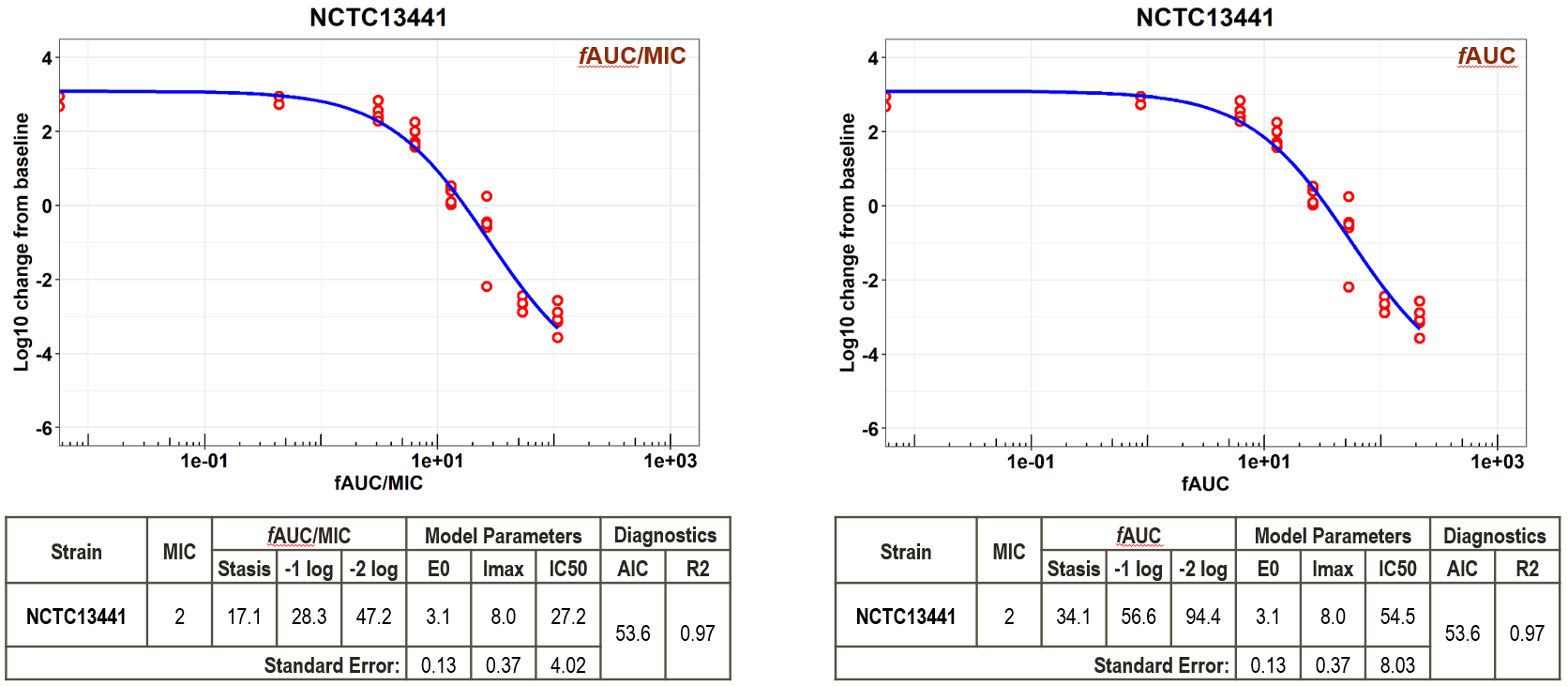


*E. coli* 997577


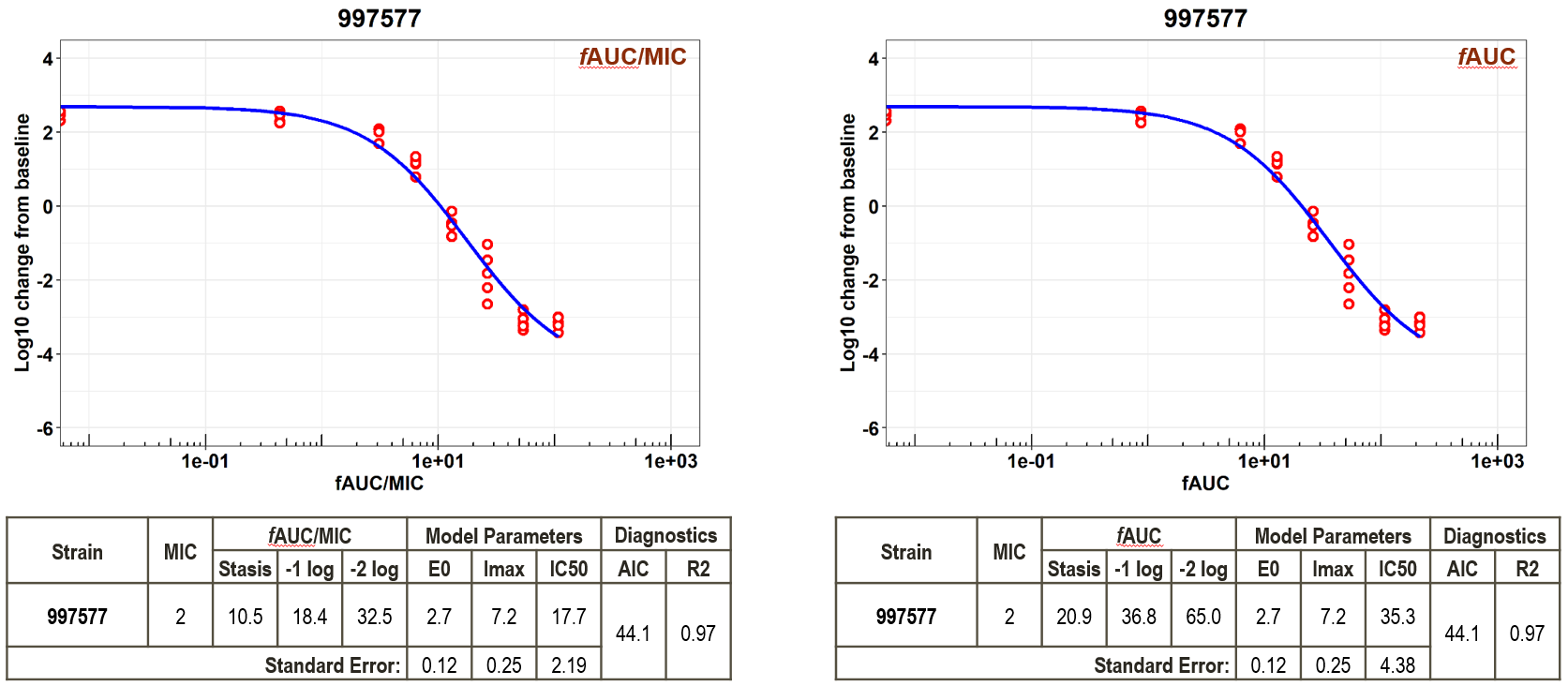


*E. coli* ALL


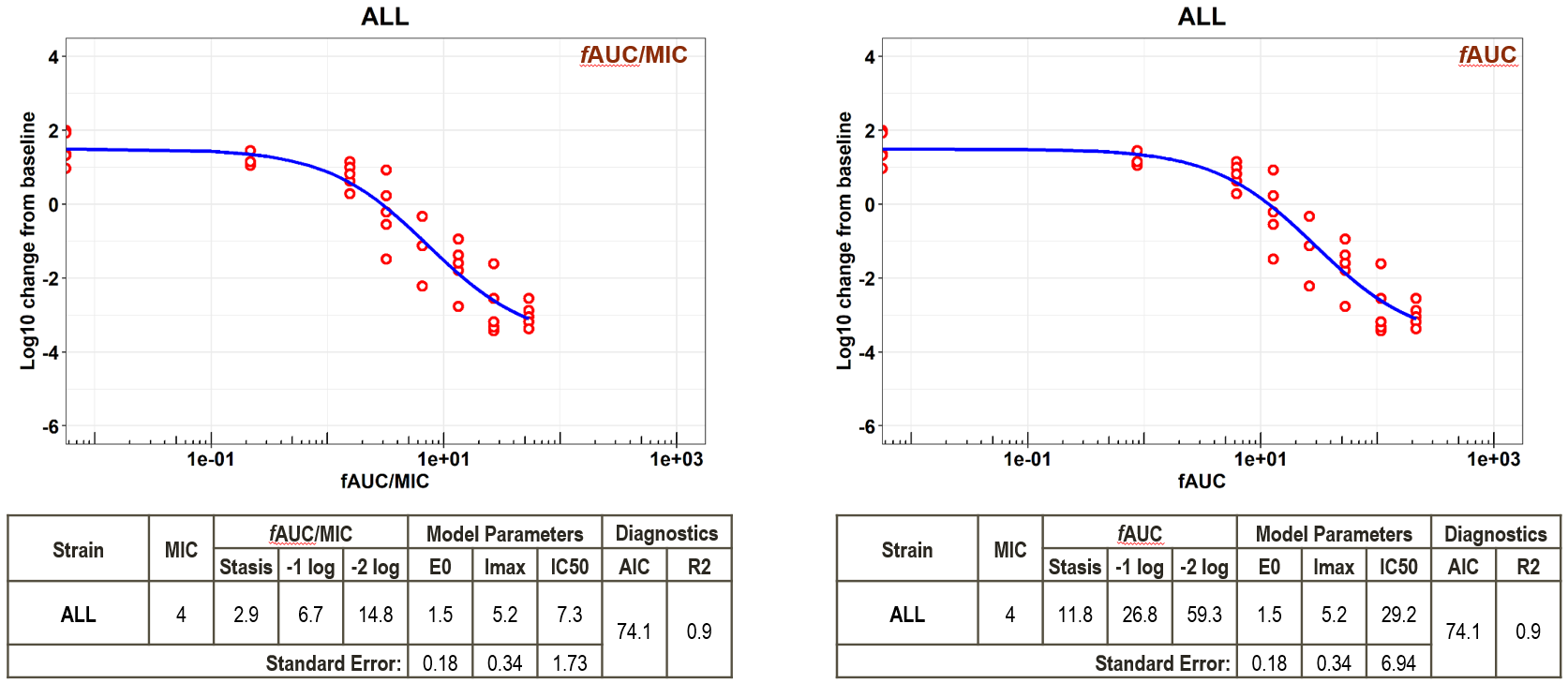


*E. coli* IR5


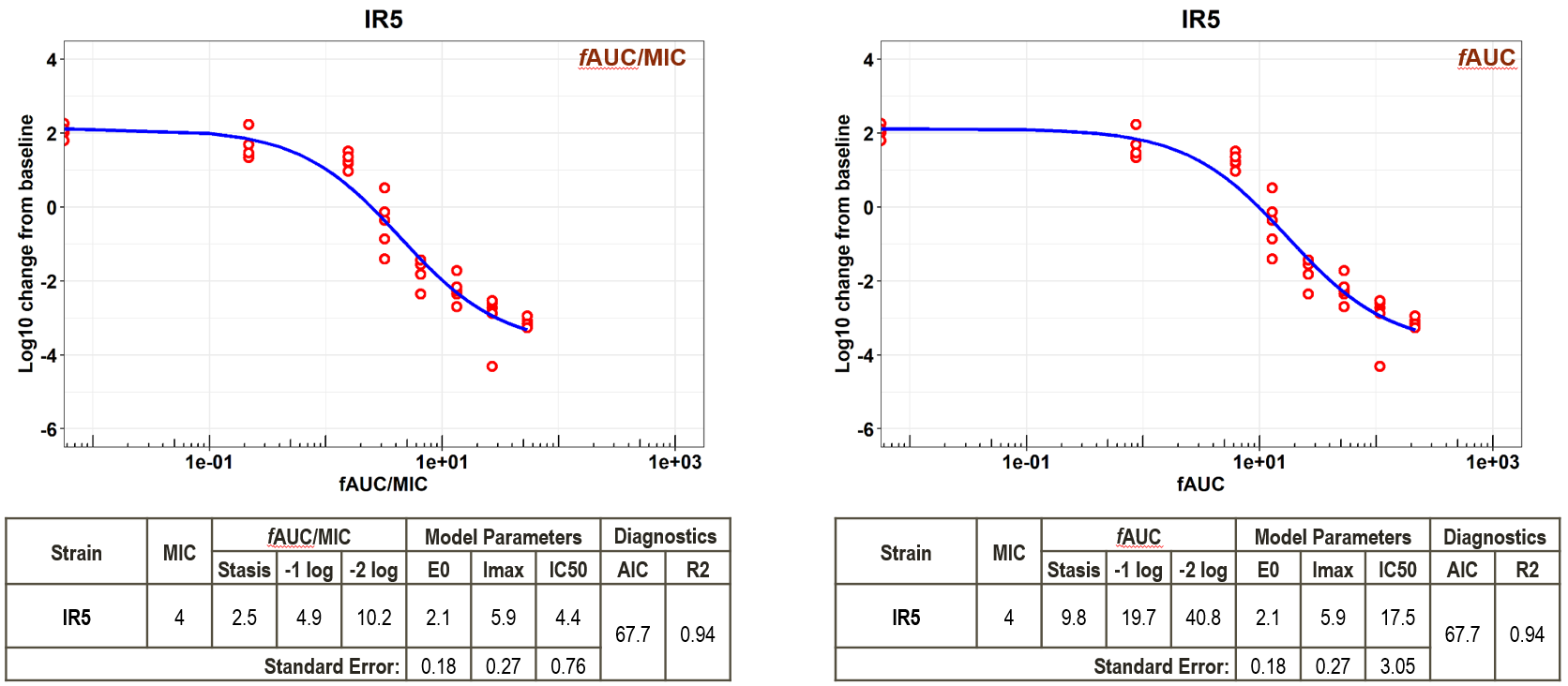


*E. coli* 1139570


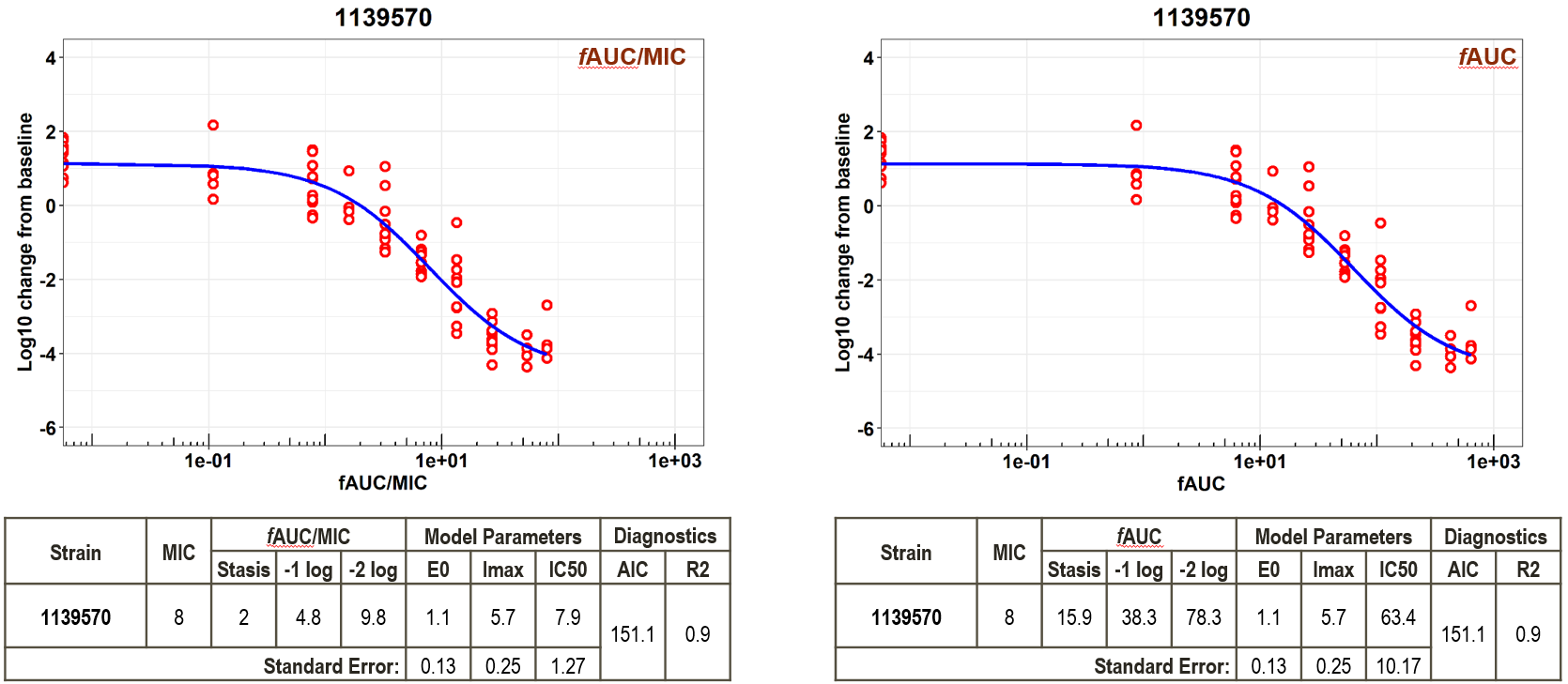


*E. coli* 774319


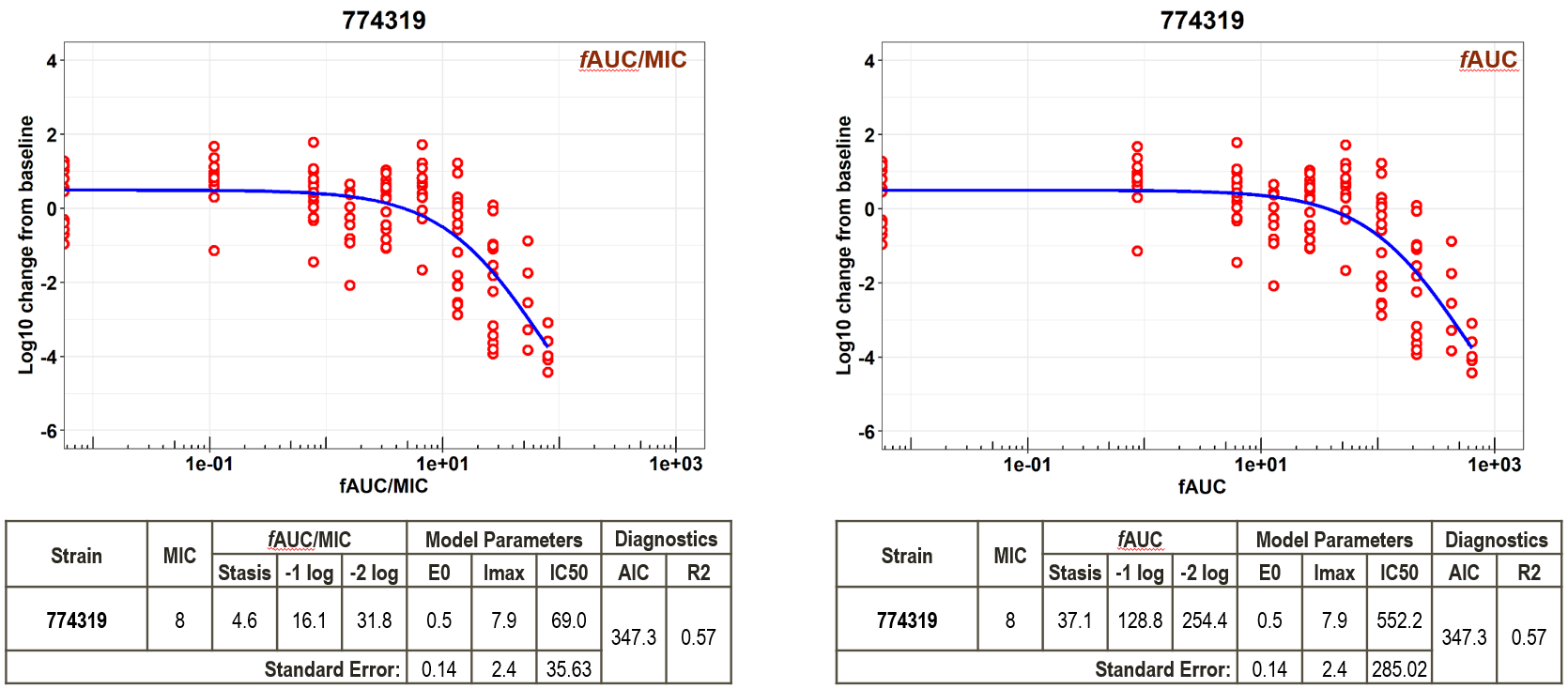


*E. coli* 817317


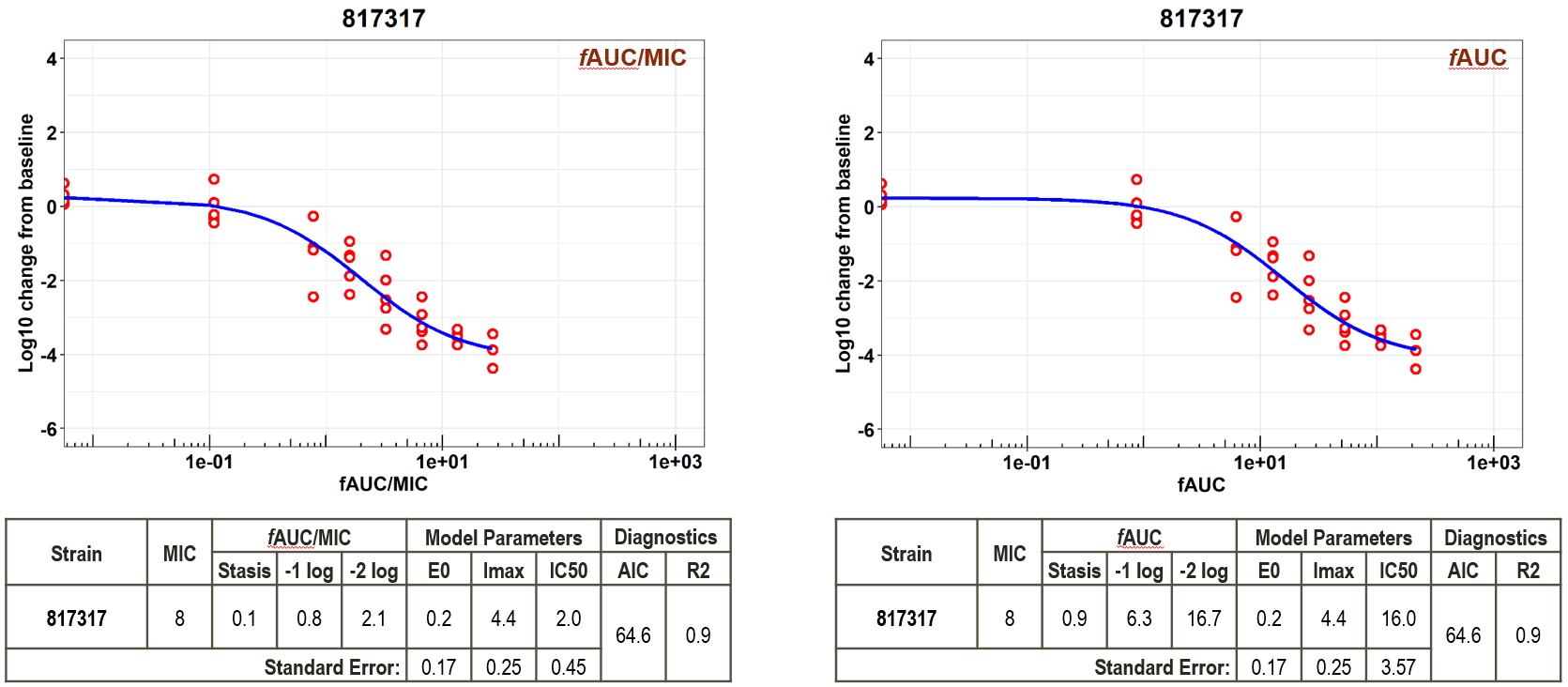


*E. coli* 771034


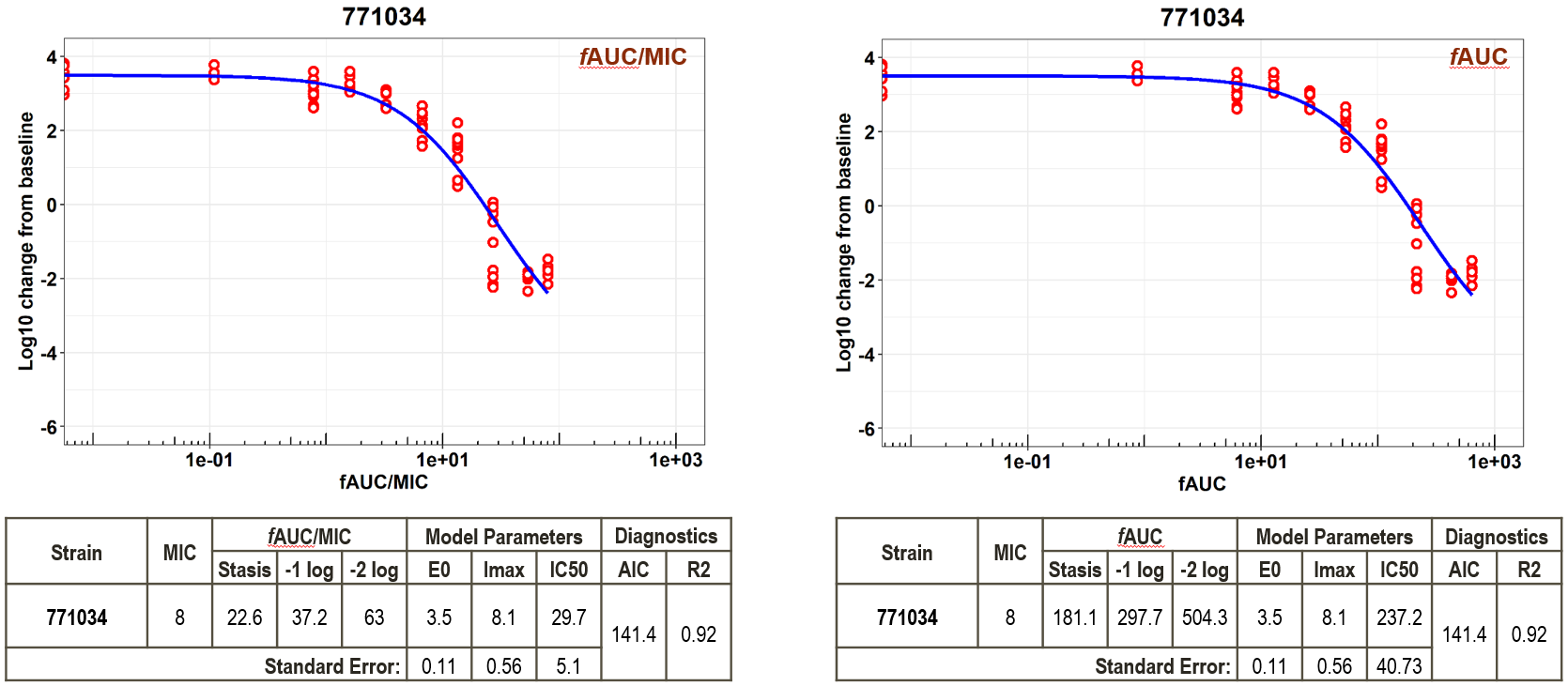


*E. coli* 764023


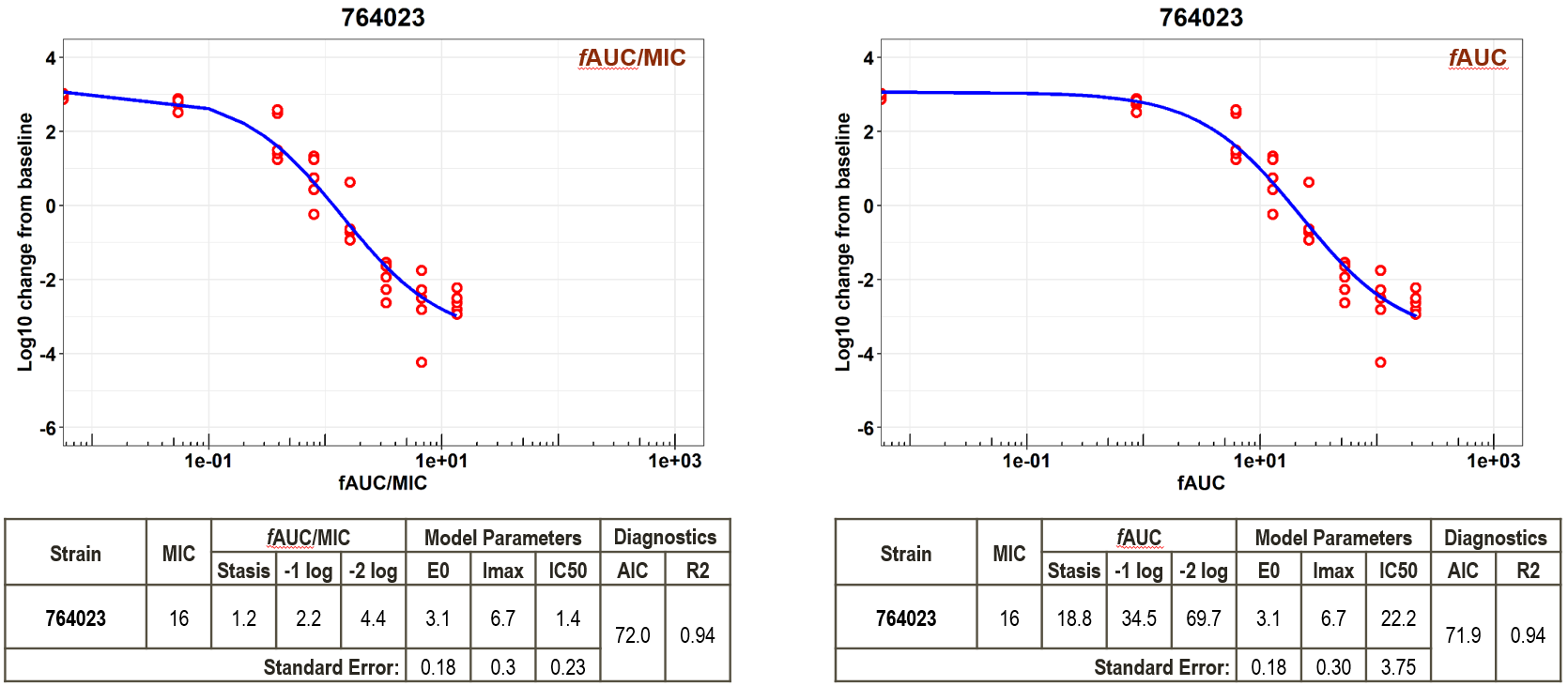


*E. coli* 1032890


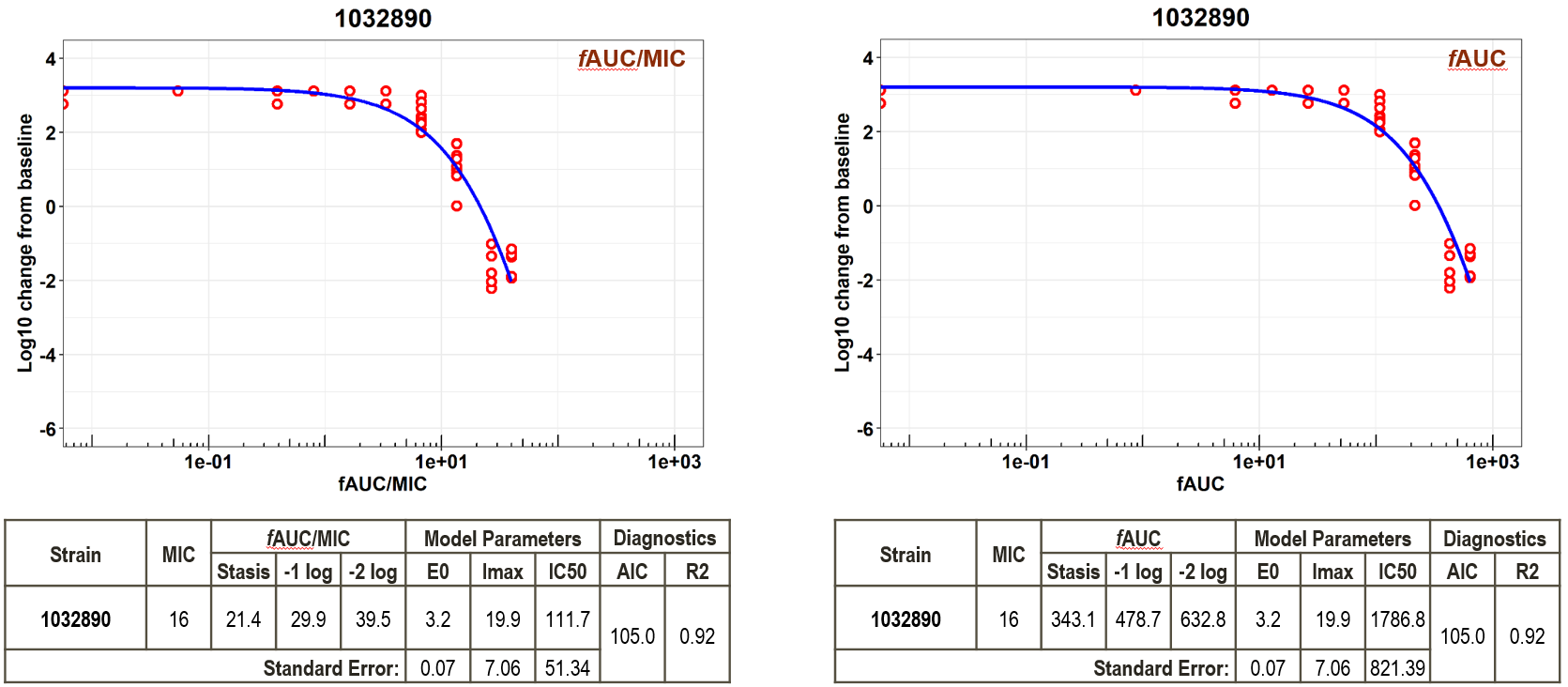


*E. coli* 823196


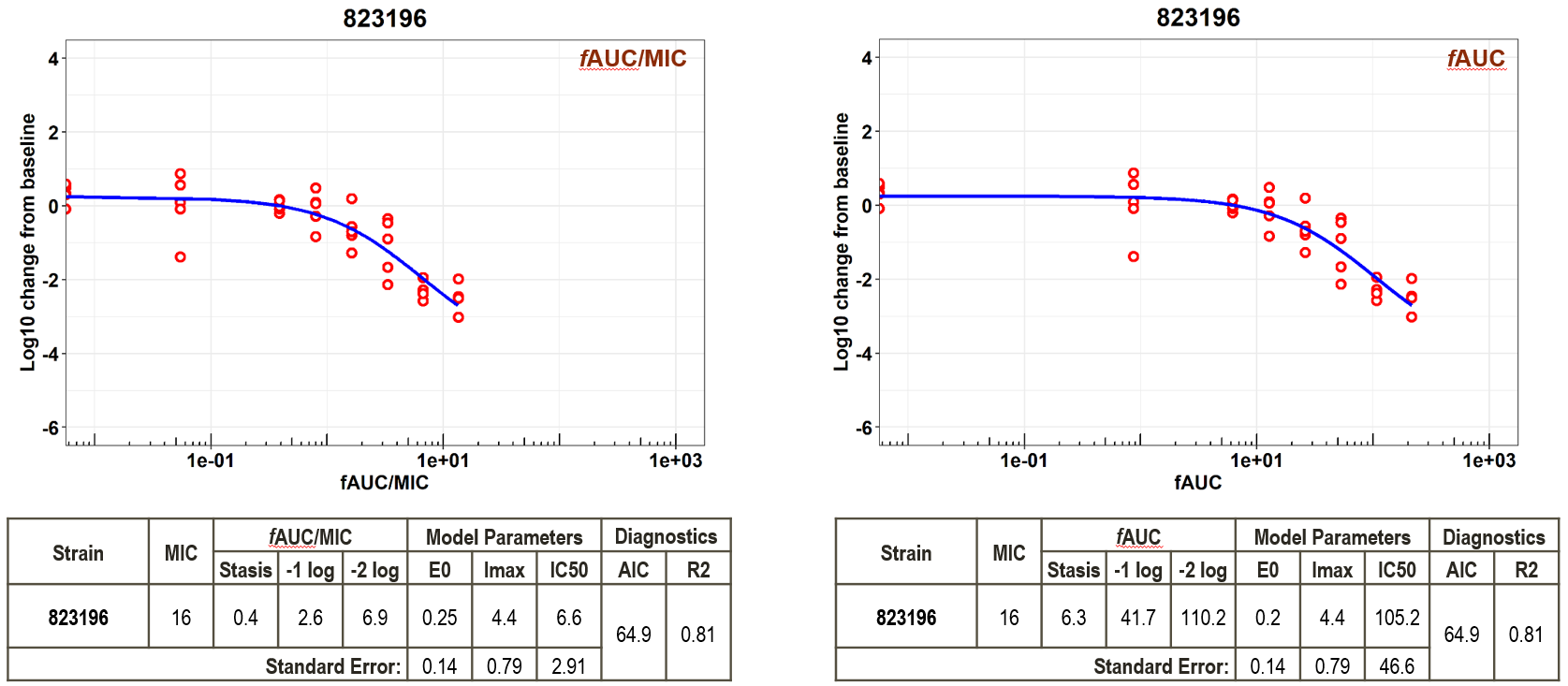


*E. coli* 718513


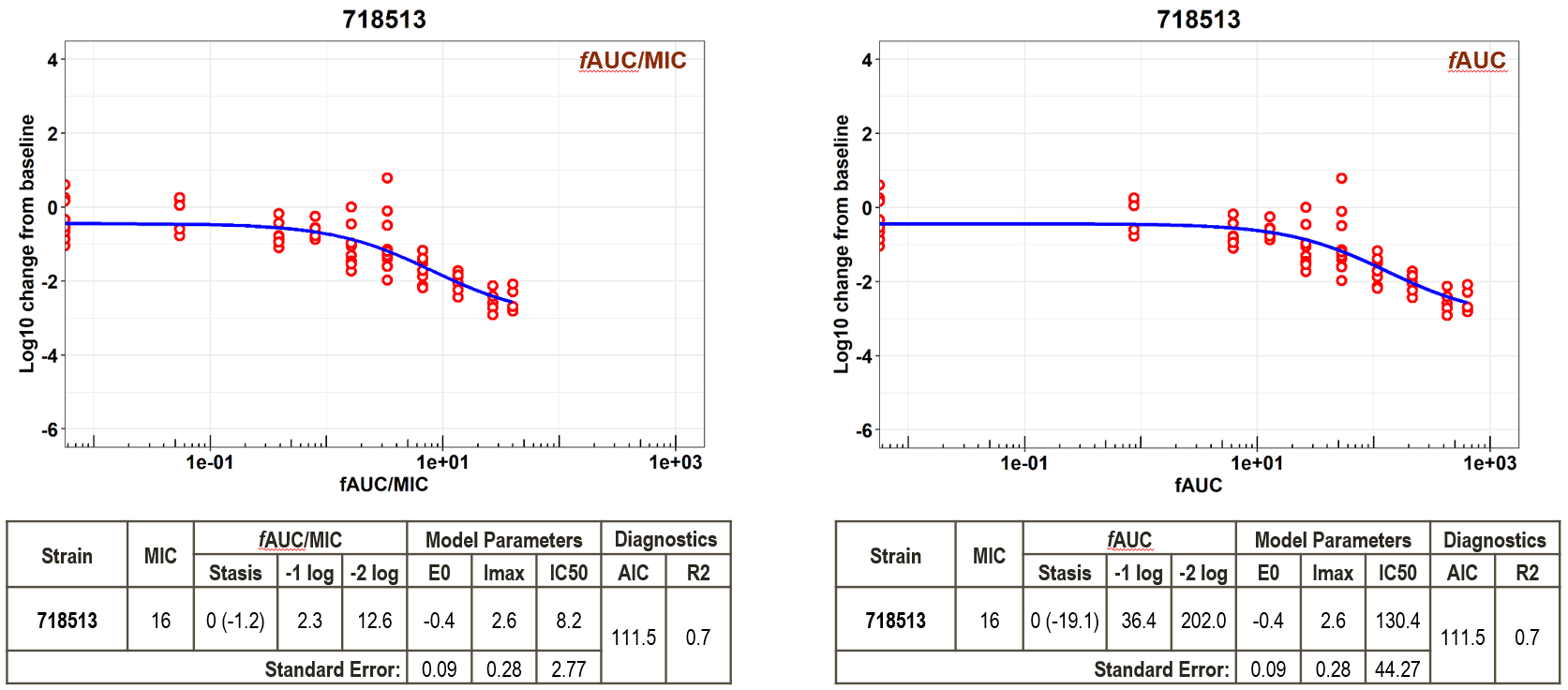


**Figure S9:** Exposure-response relationships between fAUC/MIC ratio or fAUC (not normalized by MIC) for gepotidacin and change in bacterial burden from baseline for 7 *K. pneumoniae* isolates tested in a neutropenic thigh infection model in mice; colored symbols represent datapoints for individual mice; solid line represents the fitted model with tabulated model parameters, diagnostics and calculated PK/PD targets; some isolates were tested in multiple studies, and only the pooled data was analyzed

*K. pneumoniae* 1286210


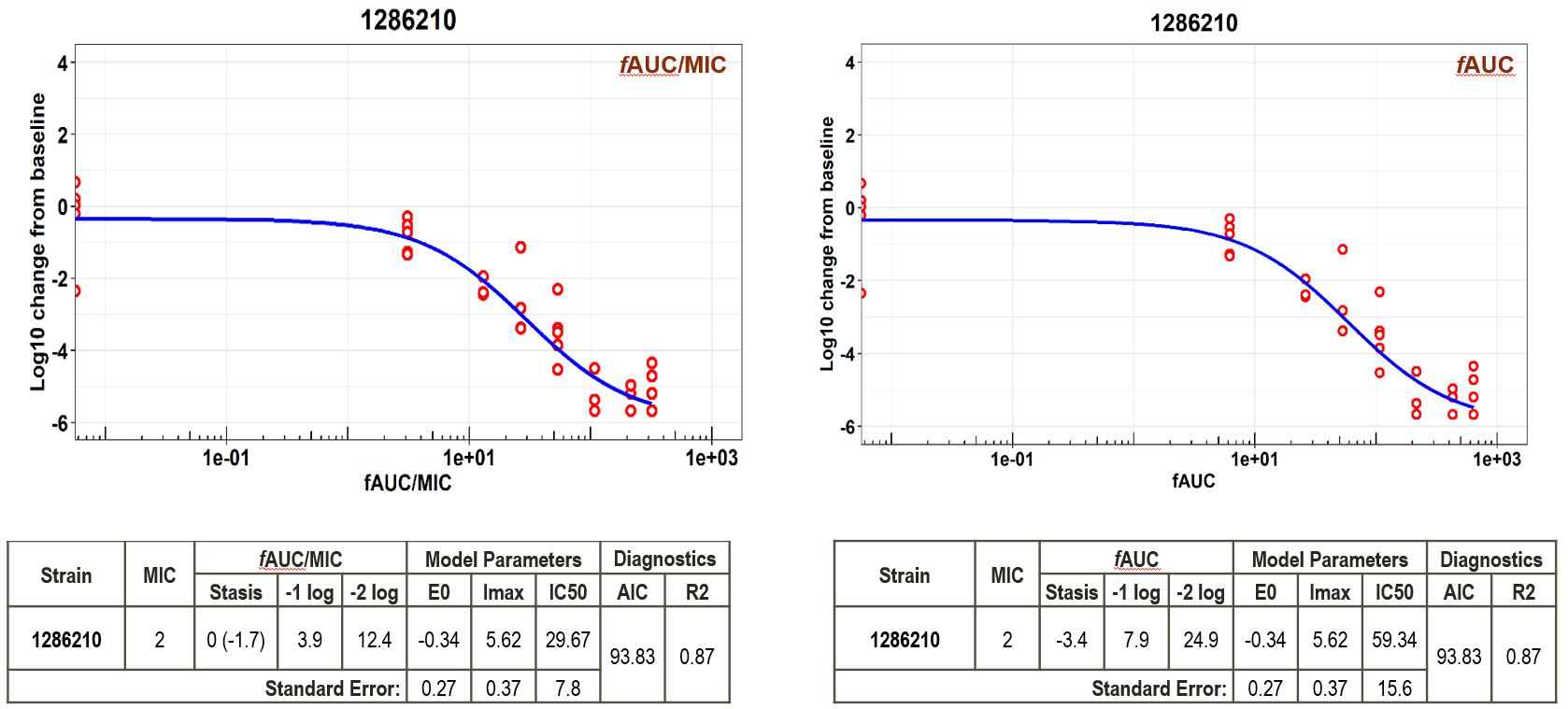


*K. pneumoniae* 1478575


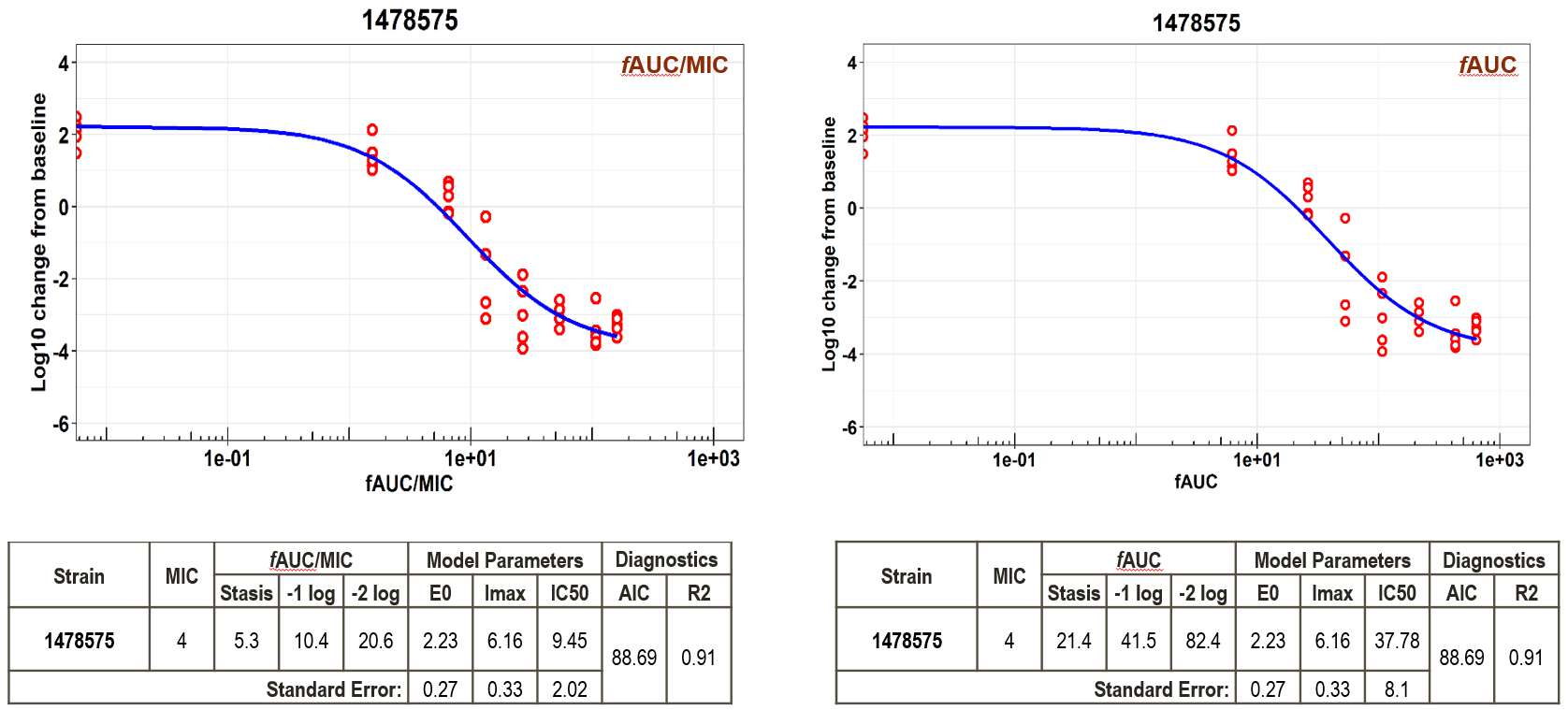


*K. pneumoniae* 1478677


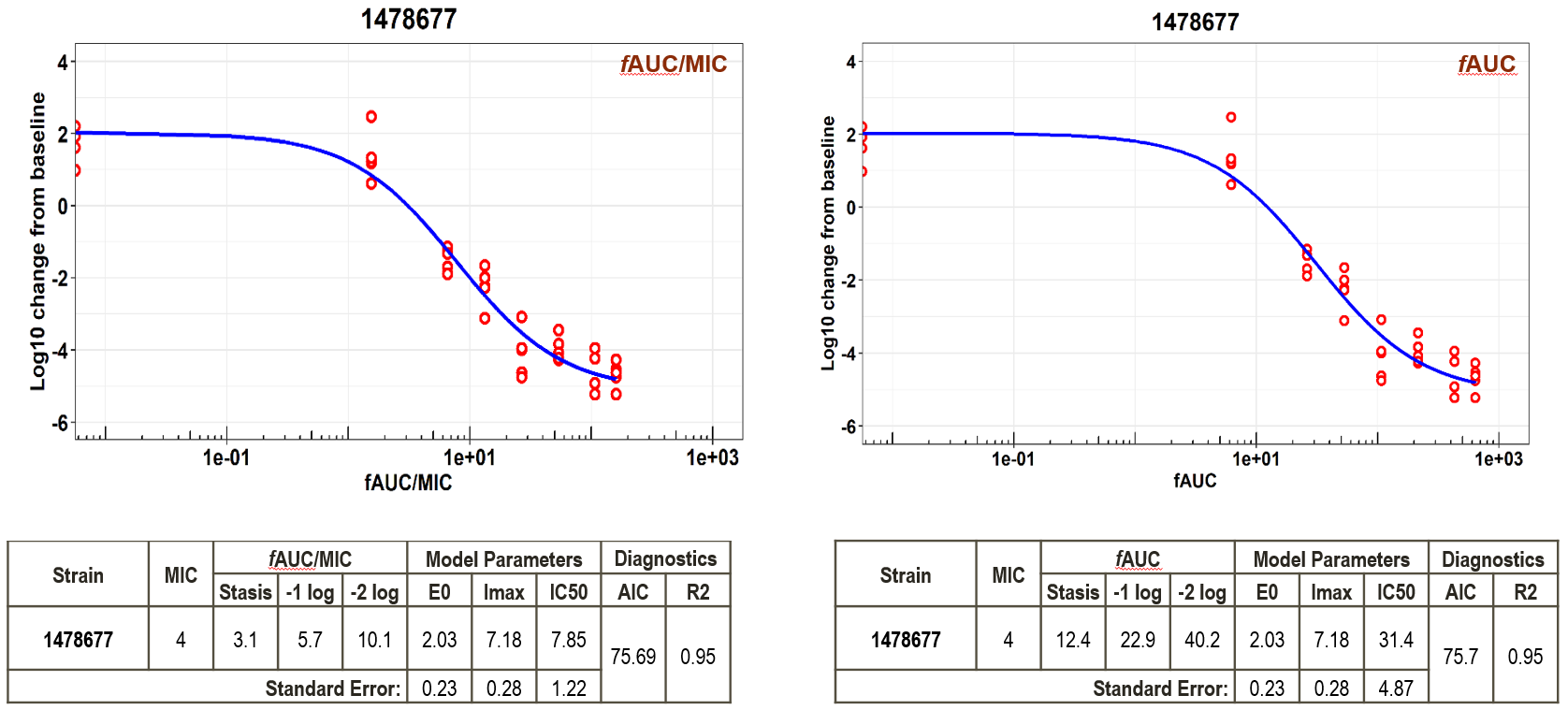


*K. pneumoniae* 1203214


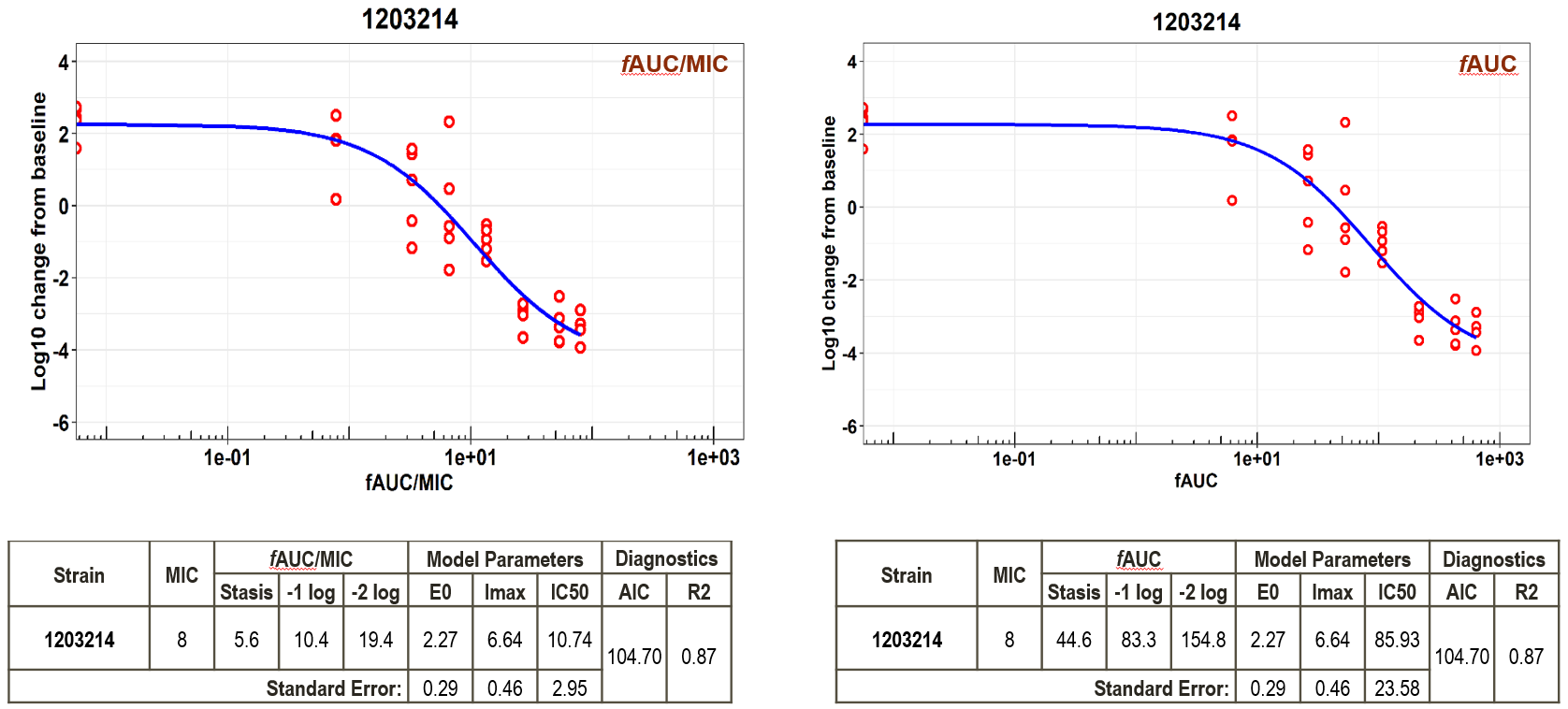


*K. pneumoniae* 1449616


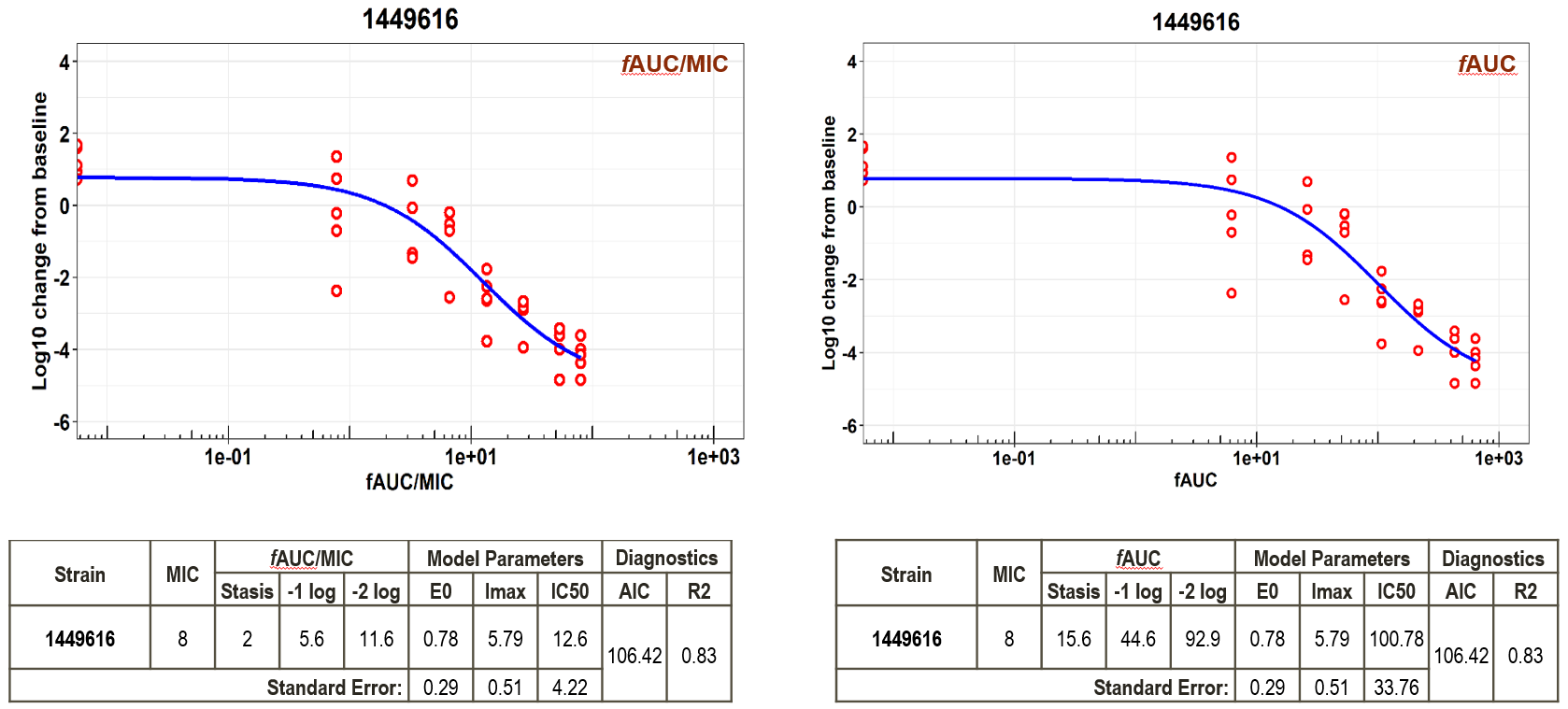


*K. pneumoniae* 1511191


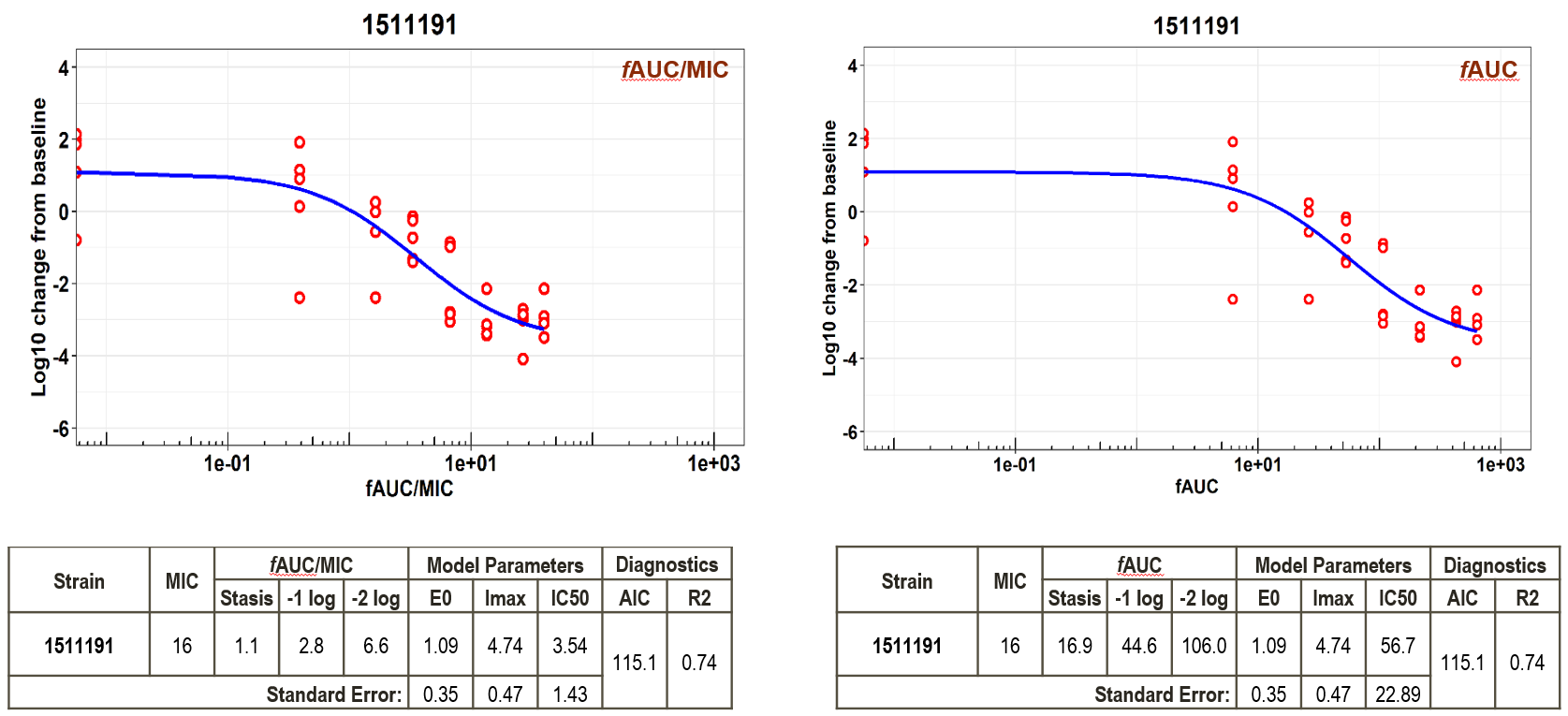


*K. pneumoniae* 1511289


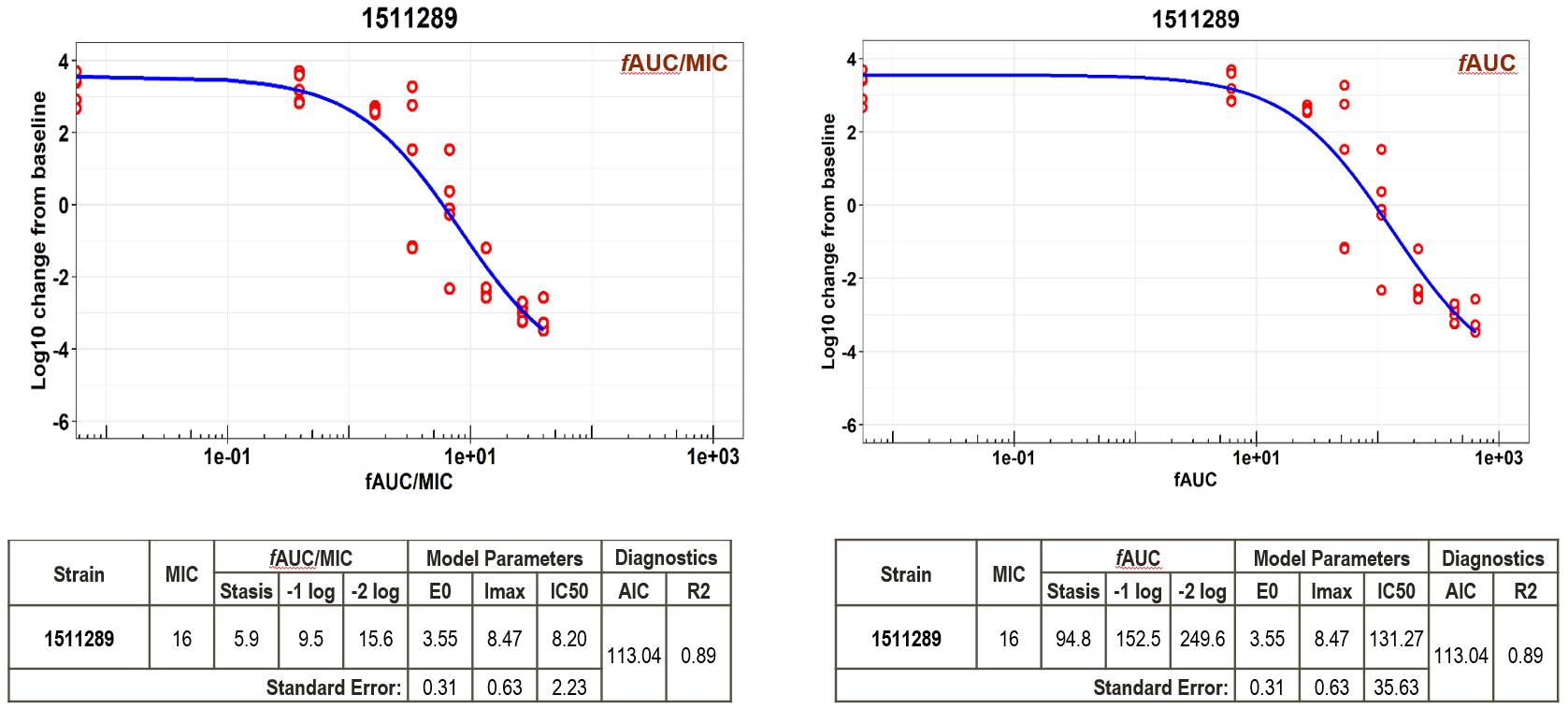


**TABLES:**

**Table S1:** Final gepotidacin population PK model parameter estimates following subcutaneous administration to mice

| **PK parameter** | **Estimate** | **RSE (%)** |
| --- | --- | --- |
| CLpop (L/h) | 0.101 | 4 |
| Vpop (L) | 0.018 | 13 |
| Kapop (1/h) | 0.886 | 4 |
| Dose effect on Kapop | -0.437 | 8 |
| IIV on CL | 38.9% | 16 |
| IIV on Ka | 20.3% | 46 |
| Add err (mg/L) | 0.128 | 27 |
| %CV for exponential models = [exp(variance)-1]^1/2 * 100 | | |
| %RSE= (Standard error of estimate/Estimate) *100 | | |

**Table S2:** Free-drug gepotidacin post-hoc PK parameters (fAUC_0-24h_ and fCmax) obtained by non-compartmental analysis for total daily exposure based on dosing four times within 24h at 6-hourly intervals

| **Dose (mg/kg)** | ***f*AUC_0-24h_ (µg.h/ml)** | ***f*Cmax (µg/ml)** |
| --- | --- | --- |
| 0 | 0.00 | 0.00 |
| 1 | 0.87 | 0.56 |
| 6.25 | 6.20 | 2.49 |
| 12.5 | 12.78 | 4.10 |
| 25 | 26.13 | 6.58 |
| 50 | 53.09 | 10.34 |
| 100 | 107.28 | 16.88 |
| 200 | 215.41 | 27.42 |
| 400 | 428.79 | 44.73 |
| 600 | 638.08 | 60.04 |

**Table S3:**  Free-drug gepotidacin post-hoc PK parameters (%fT>MIC) obtained by non-compartmental analysis for total daily exposure based on dosing four times within 24h at 6-hourly intervals

| **Dose**  **(mg/kg)** | **Gepotidacin MIC (μg/mL)** | | | | | | |
| --- | --- | --- | --- | --- | --- | --- | --- |
|  | **0.25** | **0.5** | **1** | **2** | **4** | **8** | **16** |
| **0** | 0.0 | 0.0 | 0.0 | 0.0 | 0.0 | 0.0 | 0.0 |
| **1** | 7.7 | 3.3 | 0.0 | 0.0 | 0.0 | 0.0 | 0.0 |
| **6.25** | 20.8 | 16.7 | 11.7 | 5.3 | 0.0 | 0.0 | 0.0 |
| **12.5** | 29.7 | 24.2 | 18.5 | 12.3 | 2.2 | 0.0 | 0.0 |
| **25** | 42.8 | 35.5 | 28.2 | 20.5 | 12.2 | 0.0 | 0.0 |
| **50** | 62.2 | 52.3 | 42.3 | 32.5 | 22.2 | 10.5 | 0.0 |
| **100** | 90.5 | 77.2 | 64.0 | 50.5 | 37.0 | 23.0 | 5.8 |
| **200** | 100 | 100 | 95.9 | 77.8 | 59.8 | 41.4 | 22.3 |
| **400** | 100 | 100 | 100 | 100 | 94.7 | 70.2 | 45.4 |
| **600** | 100 | 100 | 100 | 100 | 99.9 | 94.1 | 64.8 |

**Table S4:** Diagnostics for PK/PD model selection based on co-modeling data across all the isolates for each bacterial species

| **PK/PD Index** | ***E. coli* (17 strains)** | | | ***K. pneumoniae* (7 strains)** | | |
| --- | --- | --- | --- | --- | --- | --- |
|  | **Model parameters^a^** | **AIC** | **Adjusted r2** | **Model parameters^a^** | **AIC** | **Adjusted r2** |
| fAUC | 3 | 3838.5 | 0.35 | 3 | 965.2 | 0.70 |
| fAUC/MIC | 3 | 3363.9 | 0.61 | 3 | 849.6 | 0.80 |
|  | 4 | 3363.6 | 0.61 | 4 | 851.6 | 0.80 |
| fCmax/MIC | 3 | 3395.5 | 0.60 | 3 | 873.2 | 0.78 |
|  | 4 | 3397.5 | 0.60 | 4 | 870.4 | 0.79 |
| %fT>MIC | 3 | 3480.7 | 0.56 | 3 | 888.7 | 0.77 |
|  | 4 | 3481.9 | 0.56 | 4 | 888.4 | 0.77 |
| ^a^The three parameters in the inhibitory Imax model were E0, Imax, and IC50 (with slope, or gamma, set as a constant value of 1). The 4-parameter version of the model also included gamma as a variable. | | | | | | |

**Table S5:** Summary and statistics of *f*AUC/MIC required for gepotidacin to achieve stasis, 1-log_10_ or 2-log_10_ reductions in bacterial burden compared with baseline against 17 isolates of *E. coli* (representing *E. coli* PK/PD targets) as studied in a thigh infection model in neutropenic mice

| **Strain** | **MIC (µg/mL)** | **Change in VTC^a^** | ***f*AUC/MIC ratios** | | |
| --- | --- | --- | --- | --- | --- |
|  |  |  | **Stasis** | **1-log_10_ reduction** | **2-log_10_ reduction** |
| Y6702902277A | 0.25 | +1.26 | 0.6 | 1.4 | 2.9 |
| Y6702665868B | 0.25 | +2.04 | 7.7 | 18.3 | 37.9 |
| Y6700050509B | 0.5 | +1.38 | 1.5 | 3.2 | 5.9 |
| Y6700034261B^b^ | 0.5 | +0.65 | 2.8 | 8.1 | 17.1 |
| ATCC25922 | 1 | +2.27 | 13.7 | 23.1 | 37.7 |
| NCTC13441 | 2 | +2.89 | 17.1 | 28.3 | 47.2 |
| 997577 | 2 | +2.48 | 10.5 | 18.4 | 32.5 |
| ALL | 4 | +1.52 | 2.9 | 6.7 | 14.8 |
| IR5 | 4 | +2.05 | 2.5 | 4.9 | 10.2 |
| 1139570 | 8 | +1.28 | 2 | 4.8 | 9.8 |
| 774319^b^ | 8 | +0.32 | 4.6 | 16.1 | 31.8 |
| 817317^b^ | 8 | +0.35 | 0.1 | 0.8 | 2.1 |
| 771034 | 8 | +3.30 | 22.6 | 37.2 | 63 |
| 764023 | 16 | +2.96 | 1.2 | 2.2 | 4.4 |
| 1032890 | 16 | +2.94 | 21.4 | 29.9 | 39.5 |
| 823196^b^ | 16 | +0.33 | 0.4 | 2.6 | 6.9 |
| 718513^b^ | 16 | -0.38 | 0 | 2.3 | 12.6 |
| **Summary statistics including all *E. coli* isolates** | | | | | |
| Mean ± SD | | | 6.6 ± 7.7 | 12 ± 12 | 22 ± 18 |
| 50^th^ percentile (Median) | | | 2.8 | 6.7 | 15 |
| 75^th^ percentile | | | 12 | 21 | 38 |
| Range (Min – Max) | | | 0 – 23 | 0.8 – 37 | 2.1 – 63 |
| **Subset analysis excluding *E. coli* isolates with <1 log_10_ of growth** | | | | | |
| Mean ± SD | | | 8.6 ± 8.2 | 15 ± 13 | 25 ± 20 |
| 50^th^ percentile (Median) | | | 5.3 | 13 | 24 |
| 75^th^ percentile | | | 16 | 27 | 39 |
| Range (Min – Max) | | | 0.6 – 23 | 1.4 – 37 | 2.9 – 63 |
| **Summary using 1-log_10_ reduction endpoint for *E. coli* isolates with ≥1 log_10_ of growth  and 2-log_10_ reduction endpoint for *E. coli* isolates with <1 log_10_ of growth** | | | | | |
| Mean ± SD | | | 15 ± 12 | | |
| 50^th^ percentile (Median) | | | 13 | | |
| 75^th^ percentile | | | 26 | | |
| Range (Min – Max) | | | 1.4 – 37 | | |
| ^a^Average change in colony-forming units (CFU) at end of study in vehicle-treated control (VTC) mice compared with the average baseline CFU in untreated mice at 1h post infection | | | | | |
| ^b^Strain demonstrated less than an average 1-log_10_ growth in VTC mice | | | | | |

**Table S6:** Summary and statistics of *f*AUC/MIC required for gepotidacin to achieve stasis, 1-log_10_ or 2-log_10_ reductions in bacterial burden compared with baseline against 7 isolates of *K. pneumoniae* (representing *K. pneumoniae* PK/PD targets) as studied in a thigh infection model in neutropenic mice

| **Strain** | **MIC (µg/mL)** | **Change in VTC^a^** | ***f*AUC/MIC ratios** | | |
| --- | --- | --- | --- | --- | --- |
|  |  |  | **Stasis** | **1-log_10_ reduction** | **2-log_10_ reduction** |
| 1286210^b^ | 2 | -0.33 | 0 | 3.9 | 12.4 |
| 1478575 | 4 | +2.06 | 5.3 | 10.4 | 20.6 |
| 1478677 | 4 | +1.73 | 3.1 | 5.7 | 10.1 |
| 1203214 | 8 | +2.37 | 5.6 | 10.4 | 19.4 |
| 1449616 | 8 | +1.21 | 2 | 5.6 | 11.6 |
| 1511191 | 16 | +1.26 | 1.1 | 2.8 | 6.6 |
| 1511289 | 16 | +3.22 | 5.9 | 9.5 | 15.6 |
| **Summary statistics including all *K. pneumoniae* isolates** | | | | | |
| Mean ± SD | | | 3.3 ± 2.4 | 6.9 ± 3.2 | 14 ± 5.1 |
| 50^th^ percentile (Median) | | | 3.1 | 5.7 | 12 |
| 75^th^ percentile | | | 5.6 | 10 | 19 |
| Range (Min – Max) | | | 0 – 5.9 | 2.8 – 10 | 6.6 – 21 |
| **Subset analysis excluding *K. pneumoniae* isolates with <1 log_10_ of growth** | | | | | |
| Mean ± SD | | | 3.8 ± 2.0 | 7.4 ± 3.2 | 14 ± 5.5 |
| 50^th^ percentile (Median) | | | 4.2 | 7.6 | 14 |
| 75^th^ percentile | | | 5.7 | 10 | 20 |
| Range (Min – Max) | | | 1.1 – 5.9 | 2.8 – 10 | 6.6 – 21 |
| **Summary using 1-log_10_ reduction endpoint for *K. pneumoniae* isolates with ≥1 log_10_ of growth and 2-log_10_ reduction endpoint for *K. pneumoniae* isolates with <1 log_10_ of growth** | | | | | |
| Mean ± SD | | | 8.1 ± 3.4 | | |
| 50^th^ percentile (Median) | | | 9.5 | | |
| 75^th^ percentile | | | 10 | | |
| Range (Min – Max) | | | 2.8 – 12 | | |
| ^a^Average change in colony-forming units (CFU) at end of study in vehicle-treated control (VTC) mice compared with the average baseline CFU in untreated mice at 1h post infection | | | | | |
| ^b^Strain demonstrated less than an average 1-log_10_ growth in VTC mice | | | | | |

**Table S7:** Summary statistics of AUC/MIC required for gepotidacin to achieve stasis, 1-log or 2-log reductions in bacterial burden compared with baseline against all *E. coli* and *K. pneumoniae* isolates combined, representing general Enterobacterales PK/PD targets, as studied in a thigh infection model in neutropenic mice

|  | ***f*AUC/MIC ratios** | | |
| --- | --- | --- | --- |
|  | **Stasis** | **1-log_10_ reduction** | **2-log_10_ reduction** |
| **Summary statistics including all isolates** | | | |
| Mean ± SD | 5.6 ± 6.7 | 11 ± 10 | 20 ± 16 |
| 50^th^ percentile (Median) | 2.9 | 6.2 | 14 |
| 75^th^ percentile | 7.3 | 18 | 32 |
| Range (Min – Max) | 0 – 23 | 0.8 – 37 | 2.1 – 63 |
| **Subset analysis excluding isolates with <1 log_10_ of growth^a^** | | | |
| Mean ± SD | 7.0 ± 7.1 | 12 ± 11 | 22 ± 17 |
| 50^th^ percentile (Median) | 4.2 | 8.1 | 15 |
| 75^th^ percentile | 11 | 20 | 38 |
| Range (Min – Max) | 0.6 – 23 | 1.4 – 37 | 2.9 – 63 |
| **Summary using 1-log_10_ reduction endpoint for isolates with ≥1 log_10_ of growth and 2-log_10_ reduction endpoint for isolates with <1 log_10_ of growth^a^** | | | |
| Mean ± SD | 13 ± 11 | | |
| 50^th^ percentile (Median) | 10 | | |
| 75^th^ percentile | 18 | | |
| Range (Min – Max) | 1.4 – 37 | | |
| ^a^See Tables S5 and S6 for strain growth | | | |
